# The Role of the Amygdala in Facial Trustworthiness Processing: A Systematic Review and Meta-Analyses of fMRI Studies

**DOI:** 10.1371/journal.pone.0167276

**Published:** 2016-11-29

**Authors:** Sara Santos, Inês Almeida, Bárbara Oliveiros, Miguel Castelo-Branco

**Affiliations:** 1 Visual Neuroscience Laboratory, Institute for Biomedical Imaging and Life Sciences (CNC.IBILI), Faculty of Medicine, University of Coimbra (UC), Coimbra, Portugal; 2 Laboratory of Biostatistics and Medical Informatics, Institute for Biomedical Imaging and Life Sciences (CNC.IBILI), Faculty of Medicine, University of Coimbra (UC), Coimbra, Portugal; 3 Institute for Nuclear Sciences Applied to Health (ICNAS), Brain Imaging Network of Portugal, Coimbra, Portugal; University of Toyama, JAPAN

## Abstract

**Background:**

Faces play a key role in signaling social cues such as signals of trustworthiness. Although several studies identify the amygdala as a core brain region in social cognition, quantitative approaches evaluating its role are scarce.

**Objectives:**

This review aimed to assess the role of the amygdala in the processing of facial trustworthiness, by analyzing its amplitude BOLD response polarity to untrustworthy versus trustworthy facial signals under fMRI tasks through a Meta-analysis of effect sizes (MA). Activation Likelihood Estimation (ALE) analyses were also conducted.

**Data sources:**

Articles were retrieved from MEDLINE, ScienceDirect and Web-of-Science in January 2016. Following the PRISMA statement guidelines, a systematic review of original research articles in English language using the search string “(face OR facial) AND (trustworthiness OR trustworthy OR untrustworthy OR trustee) AND fMRI” was conducted.

**Study selection and data extraction:**

The MA concerned amygdala responses to facial trustworthiness for the contrast Untrustworthy vs. trustworthy faces, and included whole-brain and ROI studies. To prevent potential bias, results were considered even when at the single study level they did not survive correction for multiple comparisons or provided non-significant results. ALE considered whole-brain studies, using the same methodology to prevent bias. A summary of the methodological options (design and analysis) described in the articles was finally used to get further insight into the characteristics of the studies and to perform a subgroup analysis. Data were extracted by two authors and checked independently.

**Data synthesis:**

Twenty fMRI studies were considered for systematic review. An MA of effect sizes with 11 articles (12 studies) showed high heterogeneity between studies [Q(11) = 265.68, *p* < .0001; I^2^ = 95.86%, 94.20% to 97.05%, with 95% confidence interval, CI]. Random effects analysis [RE(183) = 0.851, .422 to .969, 95% CI] supported the evidence that the (right) amygdala responds preferentially to untrustworthy faces. Moreover, two ALE analyses performed with 6 articles (7 studies) identified the amygdala, insula and medial dorsal nuclei of thalamus as structures with negative correlation with trustworthiness. Six articles/studies showed that posterior cingulate and medial frontal gyrus present positive correlations with increasing facial trustworthiness levels. Significant effects considering subgroup analysis based on methodological criteria were found for experiments using spatial smoothing, categorization of trustworthiness in 2 or 3 categories and paradigms which involve both explicit and implicit tasks.

**Limitations:**

Significant heterogeneity between studies was found in MA, which might have arisen from inclusion of studies with smaller sample sizes and differences in methodological options. Studies using ROI analysis / small volume correction methods were more often devoted specifically to the amygdala region, with some results reporting uncorrected p-values based on mainly clinical *a priori* evidence of amygdala involvement in these processes. Nevertheless, we did not find significant evidence for publication bias.

**Conclusions and implications of key findings:**

Our results support the role of amygdala in facial trustworthiness judgment, emphasizing its predominant role during processing of negative social signals in (untrustworthy) faces. This systematic review suggests that little consistency exists among studies’ methodology, and that larger sample sizes should be preferred.

## 1. Introduction

Faces play a key role in signaling social cues such as signals of trustworthiness from which people infer meaning, aiding in the process of decision-making in everyday life [1, 2]. In fact, decisions about others are influenced by our social interactions [3, 4] and have inherent repercussions in future outcomes. Our ability to understand the intentions and dispositions of others is therefore a core process in what is called social cognition, a mental process that underlies social interactions [5]. Previous studies showed that first impressions and in particular judgements of trust can be built based on brief facial exposures in the order of milliseconds [6, 7]. Although much evidence comes from the use of emotional expressions, trait judgements such as trustworthiness, competence and aggressiveness can result from exposure to neutral faces [8]. Importantly, it has been argued that the detection of trustworthiness signals is crucial for human survival [9]. In studies involving different measures of trait importance, different groups and relationships, trustworthiness was considered one of the most relevant traits. In fact, participants rated trustworthiness as the most essential characteristic in personality (among others such as cooperativeness, attractiveness, intelligence, etc) [10]. Trustworthiness appears to be a social facial signal of special significance, since it provides information about whether other individuals should be approached or avoided, trusted or distrusted [11]. It has been suggested that trustworthiness judgments may summarize other relevant trait inferences [12]. Also, it is worth to notice that some studies have suggested a strong correlation between the perceived trustworthiness of faces and the valence component, suggesting that trustworthiness judgments may be sufficient to model how the valence of faces is evaluated in the brain [13].

The social evaluation of faces has been addressed in functional neuroimaging (fMRI) studies [9, 11, 14, 15] and systematic reviews [12, 16]. Previous fMRI studies have suggested that facial trustworthiness is related with the activation of areas such as the amygdala, the insula and the fusiform gyrus (FG) [9, 11, 14, 15]. Mendle-Siedlecki et al. [16] have systematically looked at the neural correlates of face evaluation, with a focus in differences between linear and non-linear responses as well as between trustworthiness and attractiveness studies. Bzdok et al. [12] also focused on trustworthiness and attractiveness, and investigated the nature of overlapping brain networks. Both articles outline the involvement of the amygdala in face evaluation, such as during trustworthiness judgements. However, to our knowledge no other studies systematically and quantitatively assessed the amygdala response to facial signals of trustworthiness, such as untrustworthy and trustworthy faces, either under appraisal or under neuroeconomic interactions (e.g. Trust game, Ultimatum game) relying on trustworthiness decisions, particularly when taking in consideration fMRI methodology (e.g. ROI-based, whole-brain).

In general, the amygdala has been connected with lower-level emotional processing, particularly of negative stimuli, interacting with other subcortical and cortical structures for fast threat detection [17, 18]. Accordingly, some studies have found that the human amygdala is highly implicated when evaluating other people's intentions and affective state, by responding to social cues like fearful faces [19] and variations in eye gaze [20]. This corroborates the studies which point to an important role of this structure in the perceived trustworthiness of faces [3, 9, 21, 22] and in high-level social judgements and perception, more specifically with social, emotional and reward processing [23]. First evidences came from lesion studies with Adolphs et al. showing that patients with amygdala lesions or dysfunction were not able to judge others' trustworthiness [24]. In fact, patients with bilateral amygdala damage judged untrustworthy-looking faces as if they were more approachable and trustworthy compared to neurologically normal subjects [25, 26], a finding that is not observed in unilateral damaged patients [24]. Overall, the results show that the response of the right amygdala is diminished in clinical conditions affecting social cognition [15, 27–29].

Additionally, some fMRI studies indicate that the activity evoked in the amygdala by untrustworthy-looking faces is higher than for trustworthy-looking ones [7]. In other words, the amygdala response to faces increases with the decrease of their perceived trustworthiness, even when subjects are performing tasks that do not require explicit evaluation of faces [3, 9, 13, 30]. This increased response of the amygdala towards untrustworthy faces is sometimes described as following an ordinal quasilinear trend [3, 13], while other studies have found U-shaped, quadratic responses in this structure [13, 31] with higher responses at the extremes of the trustworthiness dimension [26, 32]. Nevertheless, a systematic review and meta-analysis of these data have not yet been performed.

In sum, the study of decision-making related to social cognition has led to several hypotheses supporting a putative role of the amygdala regarding the trustworthiness of faces. In the current study we planned to answer to the following questions: a) how does the amygdala respond to the polarity of trustworthiness signals in faces? (meta-analysis of effect sizes, MA); b) what regions are involved in face trustworthiness processing (activation likelihood estimation, ALE)?

Considering the above mentioned questions, a systematic review was conducted to address the role of the amygdala in facial trustworthiness processing, namely in the context of fMRI studies and considering the amplitude of blood oxygenation level dependent (BOLD) responses. PRISMA statements guidelines were followed [33, 34], with articles being retrieved from three databases, according to a predefined search strategy.

Importantly, additional independent factors have been shown to modulate the amygdala response and should therefore also be taken in consideration. A carefully examination of the methodology and statistical criteria of each study is therefore necessary to evaluate the putative role of the amygdala during trustworthiness judgements. For instance, differences in the fMRI approach used, such as the use of whole-brain or region-of-interest (ROI) based analyses might affect the incidence of false positives. Finally, the use of either *a priori* defined categories or of trustworthiness categories based on the responses of the participants must also be taken in account. Therefore, and considering possible sources of heterogeneity across studies, besides the employed quantitative analyses (MAs and ALE), methodological components of individual studies were considered for subgroup quantitative and descriptive analyses.

The authors therefore employ systematic and quantitative methods to clarify and to systematize results previously reported in the literature, in order sum up evidence of involvement of amygdala and other regions in the appraisal of facial trustworthiness.

## 2. Methods

### 2.1. Systematic review

#### 2.1.1. Data sources and literature search

**Fig 1:**
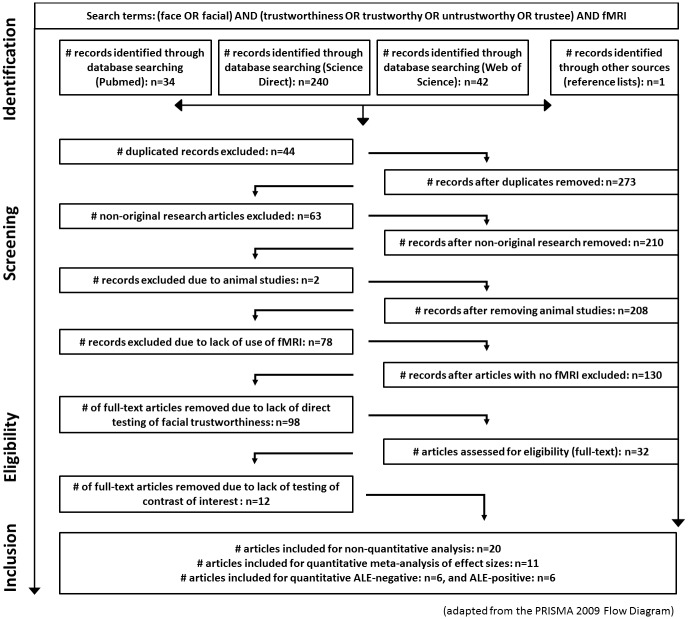
Flow diagram. Flow of information describing the different phases of the systematic review.

A systematic review was performed adhering to the principles of the PRISMA statement [33, 34]. The PRISMA statement sets steps to systematically reviewing the literature, ensuring that these reviews are performed in a standard and systematic manner. This process underlies 4 phases: identification, screening, eligibility and inclusion (Fig 1). Publications were searched on three databases, notably on MEDLINE, via PubMed (http://www.ncbi.nlm.nih.gov/pubmed), on Science Direct (Elsevier, http://www.sciencedirect.com/), and Web of Science (https://webofknowledge.com/), using the search string “(face OR facial) AND (trustworthiness OR trustworthy OR untrustworthy OR trustee) AND fMRI” (use of filter “article” and “short communication” in ScienceDirect; use of filter “article” in Web of Science). The search reported herein was undertaken in January 2016, without imposing any start and end date limit. Therefore, the search includes all the articles published until January 2016. References included in the articles deemed appropriate for full-text revision were hand-searched for retrieving other relevant publications.

#### 2.1.2. Eligibility criteria and screening phase

For a study to be considered as eligible, it had to meet the following criteria: (1) be written in English language; (2) involve adult healthy human participants (animal studies were excluded); (3) involve original research articles (e.g. review articles were excluded); (4) use of brain imaging techniques, namely functional neuroimaging (fMRI), (5) assess normal performance without introducing sources of perturbation (e.g. transcranial magnetic stimulation), (6) directly address “trustworthiness” and not other related concept, (7) test the contrasts using specifically trustworthy faces and untrustworthy faces (and not a general effect of trustworthiness). Additionally, during the screening phase, studies were considered eligible for the MA of effect sizes if they (8) make direct and separate measurements in the amygdala (e.g. without being included in a general “medial temporal lobe” label), with statistics (t, Z, r or r^2^) being reported; and for the ALE if they (9) report the Talairach or MNI coordinates (x, y, z) of the brain regions described, (10) present results of whole-brain analysis.

#### 2.1.3. Study selection and data extraction

The selection of eligible studies was performed by two authors independently (I.A. and S.S.). The reasons for rejecting the inclusion of a paper, both at this step and throughout the process of paper selection, were discussed between the authors and registered. Disagreements were solved later on by discussion until a consensus was reached. The data was collected and duplicates were eliminated (identification phase). The titles and abstracts of the remaining articles were then screened independently by the two authors (screening phase) and assessed for eligibility. All articles which were considered potentially eligible for criteria (1) to (7) by at least one of the reviewers were included for further full paper assessment (eligibility phase). These were articles presenting face stimuli in a trustworthiness task under an fMRI procedure with measurements of neural activation to both trustworthy and untrustworthy faces, testing a direct contrast between them or using linear correlation between trustworthiness values and neural activation (inclusion phase) (Fig 1).

Besides the summary statistics for the MA of effect sizes, and the brain coordinates (x,y,z) for the ALE, the following features of the included articles were extracted and summarized in S1 Table (see Supporting Information): (1) the type of task (implicit or explicit, e.g. trustworthiness judgements, age or gender categorization; no task / passive viewing) with reference to stimulus duration (e.g. subliminal, supraliminal), (2) stimulus type (faces: real or avatars; neutral or emotional), (3) the nature of stimuli presentation (static pictures or dynamic videos); (4) type of task paradigm (block or event-related design); (5) baseline condition; (6) response type and details; (7) participants characterization (sample size; gender, age); (8) data acquisition (MR system and power; sequence parameters); and (9) data analysis (standard brain template—Talairach, MNI; software of analysis; smoothing).

Finally, data were extracted by two authors (I.A. and S.S.), checked independently by each one whenever doubts occurred, and followed by a consensus decision. Importantly, authors of the articles included were contacted to clarify experimental design [35], methods [36] or to provide numerical results as only graphical ones were available [28, 32]. All responded. Gordon et al. [35] clarified that the study was event-related, Tsukiura et al. [36] clarified which regions were treated under small volume correction analysis, and both Pinkham et al. [28] and Freeman et al. [32] provided numerical data of statistical tests and results only graphically presented in their publications (see S3 and S6 Tables).

### 2.2. Data analyses

This review provides both quantitative (MA, subgroup analysis, and ALE) data analysis and non-quantitative (descriptive) summaries of neuroimaging (fMRI) findings and of the methodology used. The list of articles included in the MAs of effect sizes and ALEs can be seen in Table 1 and S2 Table.

**Table 1:** Included articles. List of articles included in the systematic review and meta-analyses (MA and ALE).

#	Articles	Articles with studies included in MA	Articles with studies included in ALE U > T	Articles with studies included in ALE T > U
1	Baron et al., 2011	x		
2	Bos et al., 2012	x		
3	Doallo et al.,2012	x	x	
4	Engell et al., 2007	x	x	
5	Freeman et al., 2014	x		
6	Gordon et al., 2009	x		x
7	Killgore et al., 2013	n.r.d.		x(*)
8	Kim et al., 2012	x		
9	Kragel et al., 2015			
10	Mattavelli et al., 2012			
11	Pinkham et al., 2008a			
12	Pinkham et al., 2008b	n.a.s.		
13	Platek et al., 2008	x	x	x
14	Rule et al., 2013			
15	Ruz et al., 2011	n.r.d.	x	x
16	Said et al., 2009	x	x	x
17	Todorov et al., 2008	x		
18	Tsukiura et al., 2013	n.r.d.		
19	van Rijn et al., 2012			
20	Winston et al., 2002	x	x	x

ALE, Activation likelihood estimation; n.a.s., no available statistical values at the time of the meta-analysis computation; n.r.d., no regions displayed; U, untrustworthy, T, trustworthy.

(*) null findings.

#### 2.2.1. Quantitative analyses: meta-analysis of effect sizes

Inclusion criteria for MA were studies using whole-brain, ROI-based and small volume correction analyses, whether applying correction for multiple comparisons or not. Moreover, in order to prevent bias in the results, even studies that did not reach statistical significance after correction or were underpowered were included. Studies presenting contrasts of untrustworthy faces versus baseline [27, 29, 37]; nonlinearities (e.g. quadratic models—see Table 2) [22, 32, 38]; p-values only or graphical information with no available t, Z or r statistical values [28]; that did not report statistics regarding non-significant contrasts within statistical maps [36, 38]; or that did not report amygdala activity [39] were automatically excluded from the quantitative MA (see Table 1 and S2 Table).

**Table 2:** Studies with linear and quadratic response models. Type of response model (Linear, Quadratic) which best fitted amygdala activation for faces in the continuum ‘Untrustworthy—Trustworthy’. Only studies presenting linear models were included in the meta-analysis of effect sizes.

Number	Author	Year	R Amygdala
1	Baron et al.	2011	**Linear**
2	Bos et al.	2012	(Linear)
3	Doallo et al.	2012	(Linear)
4	Engell et al.	2007	**Linear**
5	Freeman et al.	2014	Linear and Quadratic*
6	Gordon et al.	2009	**Linear**
7	Killgore et al.	2013	**Quadratic**
8	Kim et al.	2012	**Linear**
9	Kragel et al.	2014	-
10	Mattavelli et al.	2012	Linear** **and Quadratic**
11	Pinkham et al.	2008a	-
12	Pinkham et al.	2008b	(Linear)
13	Platek et al.	2008	**Linear**
14	Rule et al.	2013	Quadratic***
15	Ruz et al.	2011	(Linear)
16	Said et al.	2009	**Linear and Quadratic**
17	Todorov et al.	2008	**Linear**
18	Tsukiura et al.	2013	-
19	van Rijn et al.	2012	-
20	Winston et al.	2002	(Linear)

R Amygdala, right amygdala; “(linear)” means that a linear contrast was performed; “linear” in bold means that a correlation was tested instead.

* For Experiment 1 (block-design), R amygdala presented both Linear and Quadratic significant responses, while for Experiment 2 (event-related) only a quadratic response was observed.

** It concatenates R and L amygdala into one (no specific values of R amygdala).

*** It concatenates R and L amygdala into one (reports only values of concatenated bilateral amygdala ROIs, with no specific values of R amygdala).

After considering these inclusion and exclusion criteria, a MA was undertaken with statistics resulting from the specific contrast ‘Untrustworthy > Trustworthy faces’ or from the linear correlation ‘Untrustworthy—Trustworthy’ using determination and correlation coefficient (r). Whenever those were not available, both *t* and Z statistical values were taken from the original research articles and were considered to estimate the effect sizes (for details see Table 3 and S3 Table).

**Table 3:** Meta-analysis of effect sizes: (A) Confidence intervals (CI) for effect size (Pearson’s correlation coefficient) and (B) Test for heterogeneity. (A) Sample size, correlation coefficient (effect size transformations) and 95% CI for the contrast "untrustworthy > trustworthy" faces in the (right) amygdala. (B) Heterogeneity was assessed both with the inconsistency (I2) statistic and the Q coefficient.

**A**					
**Study**	**t score**	**Z score**	**Sample size**	**Correlation coefficient (Pearson’s r)**	**95% CI**
Baron et al., 2012	4,06		24	0,654	0,341 to 0,837
Bos et al., 2012	0,27		16	0,072	-0,439 to 0,548
Doallo et al., 2012		3,59	12	0,998	0,994 to 1,000
Engell et al., 2007	6,83		14	0,892	0,686 to 0,966
Freeman et al. (a), 2014	1,19		15	0,313	-0,237 to 0,711
Freeman et al. (b), 2014	0,25		15	0,069	-0,459 to 0,562
Gordon et al., 2009		-2,10	6	-0,971	-0,997 to -0,749
Kim et al., 2012		2,62	12	0,989	0,962 to 0,997
Platek et al., 2008		2,61	11	0,989	0,958 to 0,997
Said et al., 2009	2,94		32	0,473	0,149 to 0,705
Todorov et al., 2008	2,56		14	0,594	0,093 to 0,855
Winston et al., 2002		4,29	12	1,000	0,999 to 1,000
Total (fixed effects)			183	0,818	0,758 to 0,865
Total (random effects)			183	0,851	0,422 to 0,969
**B**					
**Test for heterogeneity**					
Q					265,68
DF					11
Significance level					*p* < 0,0001
I^2^ (inconsistency)					95,86%
95% CI for I^2^					94,20 to 97,05

CI, confidence interval; Measures of Inconsistency: I^2^, Inconsistency, Q, Cochran Q coefficient; DF, Degrees of Freedom; (a) supraliminal experiment; (b) subliminal experiment.

Given Student’s t score and z scores as an effect size measure, a common effect size measure was derived using the usual transformations for testing significance of Pearson’s correlation coefficient either through a Student’s t-test (1) or a Z test by the Fisher’s transformation (2), as follows:

$$r=\frac{t}{\sqrt{n-2+t^{2}}}$$ (1)

$$r=\frac{e^{2z}-1}{e^{2z}+1}=\text{tanh}\left(z\right)$$ (2)

Thereby, it was possible to have a common effect size measure to analyze, and thus perform a meta-analysis. As studies reported effect sizes by means of t or z scores, we may propose either a t and Z score by applying the inverse of eqs (1) and (2) formulas (formulas (3) and (4)) to the final effects model index:

$$t=r\sqrt{\frac{n-2}{1-r^{2}}}$$ (3)

$$r=\frac{1}{2}ln\left(\frac{1+r}{1-r}\right)=arctanh\left(r\right)$$ (4)

Heterogeneity was assessed both with the inconsistency (*I*^*2*^*)* statistic and the Q coefficient. The I^2^ Index is a standard test that measures the degree of inconsistency across studies. This test results in a range from 0% to 100%, which describe the proportion of variation in treatment effect estimates due to inter-study variation [40]. It may be interpreted as the proportion of total variance in the estimates of treatment effect that is due to heterogeneity between studies and thus it has a similar concept to the intraclass correlation coefficient in cluster sampling [41]. The Q coefficient was also used to calculate the homogeneity of effect sizes [42]. A global index about the effect’s magnitude should then be derived either from a fixed-effects model or from a random effects model [41]. If the studies only differ by the sampling error (*I*^*2*^ *< 50%*, homogeneous case), a fixed-effects model is applied in order to obtain an average effect size. If the studies’ results differ by more than the sampling error (*I*^*2*^ *> 50%*, heterogeneous case) a random-effects model is preferred instead [42]. Quantitative meta-analysis was performed in order to access heterogeneity between studies and thus, to obtain a global measure of effect which summarizes effect measures reported in individual studies. This last one may be merely indicative whenever the amount of heterogeneity is high and the number of studies is small, as is the case we studied, and must therefore be complemented with individual effect sizes and their respective confidence interval.

All the estimates included were recomputed from original articles descriptions, potentially resulting in slightly different values. All reported *p*-values are 2-tailed and analyzed at a significance level of 5%. Meta-analysis was performed with the software package MedCalc (R) (version 12.7.2.0–64 bit, Copyright 1993–2013, MedCalc Software bvba, Mariakerke, Belgium).

#### 2.2.2. Quantitative analyses: subgroup analysis

A subgroup analysis was performed by considering methodological options of experimental design, acquisition and analysis parameters of each study (for a list of factors see section 2.1.3, and for a detailed characterization see S1 and S4 Tables). Nine criteria (experimental design: (1) paradigm, (2) type of categorization; acquisition: (3) software of analysis, (4) echo time, (5) repetition time, (6) type of sequence; analysis: (7) correction for multiple comparisons, (8) smoothing, (9) contrast) were considered to group the articles/studies (S4 Table).

#### 2.2.3. Quantitative analyses: activation likelihood estimation (ALE)

ALE is a voxel-based method implemented to find convergence across functional neuroimaging experiment coordinates [43], and was performed to asses if there were consistent functional activations present in the studies evaluating the trustworthiness from faces.

Since ALE can only be performed with explicitly reported coordinates of the activated areas, only studies presenting data reported in standard stereotactic coordinates (either Talairach or MNI) were considered for the voxel-level quantitative meta-analysis [44] (studies performed using contrasts considered in this systematic review but presenting null results were nevertheless included, but with no data regarding the coordinates). We excluded studies presenting results where main effects analyses were restricted to *a priori* defined ROIs or using small volume correction, with unobtainable coordinates, data with nonspecific contrasts relative to baseline or tasks not evaluating trustworthiness [12, 45] (see S2 Table). For this analysis, data with uncorrected p-values were considered, using only results of adult healthy control (HC) groups (see Table 1, S2 and S5 Tables).

Two separate ALE meta-analyses were conducted with coordinates resulting from: (1) a negative correlation between neural responses to faces and trustworthiness (i.e., increase of the neural response with the decrease of trustworthiness levels) and (2) a positive correlation between neural responses to faces and trustworthiness (i.e., increase of the neural response with the increase of trustworthiness levels).

ALE meta-analyses were performed in Talairach space, using GingerALE 2.3 (http://www.brainmap.org/ale). Anatomical coordinates reported in MNI space were converted to Talairach space using the Lancaster (icbm2tal) transformation [46]. In ALE analysis, all foci reported for each experiment are modeled as the center of a Gaussian probability distribution. In order to model the spatial uncertainty of each focus, this approach takes into account the inter-subject and inter-laboratory variability observed in neuroimaging studies by adjusting the width of the smoothing Gaussian kernel. The information of individual foci is then merged, taking the voxel-wise union of their probability values. As a result, a modelled activation map is calculated by finding the union [47] or the maximum [48] across each Gaussian focus. The final ALE image corresponds to the union of each individual modelled activation maps [49].

Regarding this analysis, the obtained ALE maps were thresholded using 1000 permutations, *p* < .001 as cluster-forming threshold and *p* < .05 for cluster-level inference [49]. The cluster statistics identified ALE clusters, providing the coordinates of the weighted center-of-mass and peak locations, and anatomical labels were assigned by the Talairach Daemon [50].

The results are reported in accordance with the PRISMA guidelines on reporting of systematic reviews and meta-analyses [33].

#### 2.2.4. Non-quantitative analysis

The studies or results which could not be included in the quantitative statistical meta-analyses (MA and ALE) were nevertheless considered for a non-quantitative analysis. In this analysis, we reviewed the results regarding amygdala and other regions’ response to the untrustworthy vs. trustworthy face contrasts.

In addition to the quantitative subgroup analysis presented in section 2.2.2, differences in methodologic issues of each study were summarized and discussed. Importantly, *a priori* hypotheses concerning amygdala involvement in trustworthiness processing and subsequent methodology options within studies were considered.

### 2.3. Risk of bias

Assessment of risk of bias of individual studies and across studies was undertaken. In order to prevent a biased literature search in what concerns amygdala’s involvements in trustworthiness processing of facial stimuli, the “amygdala” keyword was not included as a search term. Independent assessment of articles for inclusion and data extraction was performed by two authors (I.A. and S.S.), with discussion until a consensus was achieved.

Methodological components were extracted from individual studies (S1 and S4 Tables) and used for subgroup analysis of effect sizes. Measures of variability between studies were used within the MA, and this was performed including both positive and null results of amygdala activation to the contrast Untrustworthy > trustworthy faces. Finally, only whole-brain studies were included in the ALE analysis (ROI-based and small volume correction studies were excluded).

In order to access the existence of publication bias within the meta-analysis of effect sizes, i.e. different dissemination of research findings as an effect of the nature and direction of results [51], funnel plots and Egger’s regression test of asymmetry were further performed. For the funnel plot, R software (R Studio, Version 0.99.903, RStudio, Inc.) was used, with the correlation coefficients being centered in the mean effect (normalized to “0”). Importantly, standard error of the intervention effect estimate was plotted on the vertical axis, as recommended [52]. The Egger’s regression test is used to quantify the bias captured in the funnel plot, and uses the values of the effect sizes and their precision [53].

## 3. Results

The Flow Diagram displayed in Fig 1 reflects the selection process. Our review of the literature using search items as described above identified 316 potential target articles [34 were identified via the PUBMED database, 240 through ScienceDirect and 42 via Web of Science], with 1 article being identified through other resources, namely reference lists of related articles. Forty-four articles were duplicated records, and 63 referred to non-original research articles (e.g. review, methods paper, commentary) being therefore excluded. Other reasons for exclusion were studies employing animal and not human participants (n = 2), lack of use of fMRI methodology (n = 78), and no direct assessment of trustworthiness in human faces (n = 98). A total of 32 publications were carried to full text assessment. From the identification to the eligibility phase, 285 articles were excluded, based on the information displayed in the abstracts, taking into account criteria (1) to (6) (see Methods section). Twelve additional articles were not considered in the final set as they did neither test a direct contrast between Trustworthy and Untrustworthy faces, nor tested a linear correlation with amygdala activity. The remaining 20 articles underwent quantitative (section 3.1) and non-quantitative (section 3.2) data extraction and analysis. All were published in the last 10 years, except one which dates from 2002 [25]. Characterization of the articles/studies included is detailed in S1 Table. Specifically for the quantitative analysis, the articles were incorporated in the MA of effects (sections 3.1.2 and 3.1.3) and/or in the ALE analyses (sections 3.1.5 and 3.1.6).

### 3.1. Quantitative analysis

#### 3.1.1. Meta-analysis of effect sizes: excluded studies

Given the overall inclusion criteria specifically for the quantitative MA (see section 2.1.2), nine articles and 1 study were excluded due to the fact that (a) right and left amygdala were concatenated in one single ROI resulting in conjoint statistics (2 articles: [22, 26]); (b) the contrast was performed with untrustworthy faces against baseline conditions or average trustworthiness faces (3 articles: [27, 29, 37]; 1 study: [32]); and (c) the article did not provide the values (t, Z, r or r^2^) of the contrast (4 articles: [28, 36, 38, 39]). Eleven articles (12 studies) fulfilled the criteria of inclusion in the MA.

#### 3.1.2. Meta-analysis of effect sizes: contrast ‘untrustworthy > trustworthy’ faces

An unbiased MA was performed by including also studies that were either underpowered or showed uncorrected results. Results of 12 studies from 11 articles were used to measure the amplitude of (right) amygdala responses in the contrast ‘Untrustworthy > Trustworthy’ faces.

**Fig 2:**
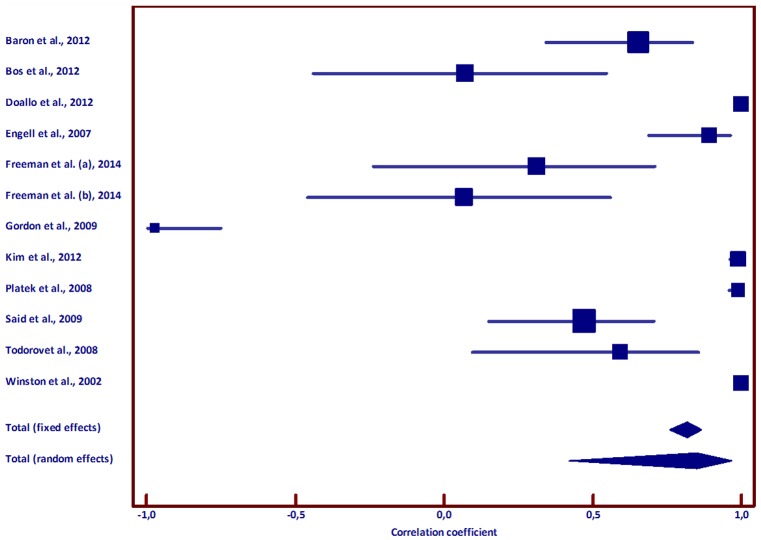
Meta-analysis of effect sizes (n = 11): Confidence intervals for effect size (Pearson’s correlation coefficient). Forest plot resulting from the meta-analysis with 12 studies (11 articles) for the contrast "Untrustworthy > Trustworthy" faces presenting central values of correlation coefficients (square markers) and their confidence intervals (horizontal lines). The size of the square markers varies with the sample size. Diamond markers represent pooled effects. The location of the diamond represents the estimated effect size and the width of the diamond reflects the precision of the estimate.

Given transformations of t and Z values, a common effect size measure to analyze was derived. As we may not assume a Z distribution since some of the studies reported t-scores, if is preferable to report the final effect size measure by means of t-scores. On the other hand, the Pearson’s correlation coefficient test usually applies the r-to-t transformation. Results shown in Table 3 and Fig 2 present right amygdala responses for ‘Untrustworthy > Trustworthy’ faces, showing a clear lateralization trend. The Cochran χ^**2**^ test (commonly known as the Q test) indicated a large amount of heterogeneity between studies (Q(11) = 265.68, *p* < .0001). However, it is usually stated that this test has poor power when few studies are being analyzed [54] and Higgins et al. suggested the use of other measures, such as the I^2^ Index [40]. For this meta-analysis, performed on 12 studies and involving 183 cases, the I^2^ Index was 95.86% (94.20% to 97.05%, with 95% confidence interval, CI), thereby confirming the large amount of heterogeneity between studies.

A global index about the effect’s magnitude of amygdala’s response to untrustworthiness was therefore derived from a random effects (RE) model [41], indicating a linear correlation (r = .851), where the lower limit for the confidence interval indicates strong correlation (r > .4) and thus a large effect size, as observed also in Fig 2 (RE(183): 0.422 to 0.969, 95% CI). Of the 12 studies (11 articles) studies considered, six resulted in a weak to moderate correlation [30–32, 55, 56], as all the other report correlations above .89 (with 95% CI above 68%).

Although random-effects can be used as a global measure of effects, given that these effects derive from a small number of studies (n = 12), with high heterogeneity, one should consider also the individual effects. Therefore, we also analyzed descriptively the studies included.

Of the 12 studies considered, all of the studies reported a negative correlation of amygdala activity with facial trustworthiness (direction untrustworthy > trustworthy), except one [35] which reported a positive correlations of amygdala with Trusting behavior, and 2 others which failed to find significance [32, 55]. Additionally, 3 studies did not report statistics associated to the outcomes of the contrast between untrustworthy and trustworthy faces, with 3 other studies reporting no differences using small volume correction [36, 38] or cluster correction [39] and 1 finding differences in the right amygdala ROI at the *p* < .05 level [28].

Regarding correlation coefficients, Freeman et al. [32] studies, both the subliminal and supraliminal tasks, and Said et al. [31] showed weaker correlations (r below .5) than the other 5 (tested in the direction untrustworthy > trustworthy faces) correlation studies. Two studies [30, 56] showed absolute values between .5 and .7. These results had a direct impact in the 95% Confidence Intervals, with only 4 studies showing CI above 90% [25, 57–59]. Large CIs were particularly found in 4 studies [30–32, 56] limiting the generalization of conclusions regarding the results of this contrast in the population.

This model showed that right amygdala responses in adult HCs are higher to untrustworthy compared to trustworthy faces.

#### 3.1.3. Meta-analysis of effect sizes: subgroup analysis

Given the heterogeneity found between studies (see above section), subgroups were generated according to methodological components taken from the experimental design, data acquisition and analysis parameters (for details concerning these factors, see Supporting Information, S1 and S4 Tables). Results showing the subgroups of studies included in the MA and in which the effect was verified are presented in a forest plot (S1 Fig) displaying all the factors and levels (groups) considered.

Statistically significant positive effects (Untrustworthy > trustworthy) were found within the groups of Smoothing “8 mm” [25, 32, 55], Task paradigm “Explicit (+implicit)” [25, 57], and for the division of Trustworthiness values in 2 to 3 categories (instead of using a Likert type scale) [55, 58]. All the remaining factors and/or levels analysed presented mainly observed positive effects, although not statistically significant, according to the expected 95% confidence interval obtained for the respective effect. Importantly, one must point that all tended to a positive effect but the large amplitude of the confidence intervals precludes a significant statistical criterion. This may be explained by the large variability within studies mainly due to their sample size.

#### 3.1.4. ALE: excluded studies

Twelve articles were excluded from the ALE analysis, due to (a) data with nonspecific contrasts relative to baseline (3 articles: [27, 29, 37]); (b) lack of reporting Talairach or MNI coordinates (1 article: [30]); (c) ROI-based or small volume correction analysis (8 articles: [26, 28, 32, 36, 37, 55, 56, 58]) (see S2 Table for a detailed list of exclusion criteria).

Two ALE meta-analysis were performed. The first analysis, concerning the negative correlation between neural responses to faces and trustworthiness, was performed with 7 studies from 6 articles. The second analysis, regarding the positive correlation between neural responses to faces and trustworthiness, was conducted with data from 6 studies retrieved from 6 articles.

#### 3.1.5. ALE: negative correlation with facial trustworthiness

**Fig 3:**
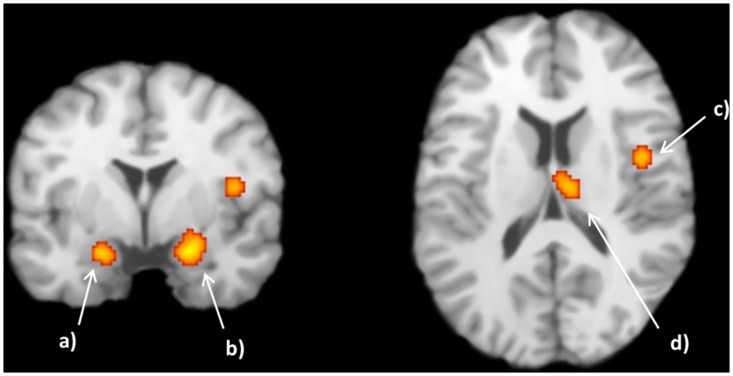
Activation likelihood estimation (ALE) meta-analysis with 7 studies (6 articles) regarding the negative correlation between neural activation and facial trustworthiness. Some of the modulated regions were a) L amygdala (-18, -4, -18), b) R Amygdala (21, -4, -17), c) R Insula, BA 13 (43, -2, 14) and d) R Thalamus (8, -15, 14). The obtained ALE maps were thresholded using 1000 permutations, *p* < .001 as cluster-forming threshold and *p* < .05 for cluster-level inference.

For the first analysis, regarding the negative value of face trustworthiness (untrustworthy > trustworthy faces), as assessed by the above mentioned contrast, activation in six clusters was found, including the right and left amygdala, the thalamus (medial dorsal nucleus) and the insula (see Fig 3 and Table 4).

**Table 4:** ALE: results for the negative correlation. Activation likelihood estimation (ALE) meta-analysis results highlighting that the amygdala is sensitive to the low face trustworthiness.

Cluster #	Volume (mm^3^)	Peak ALE value	Peak coordinates			Label
			x	y	z	
1	1384	0.015536461	24	-2	-16	R Parahippocampal Gyrus (Amygdala)
2	656	0.01224767	10	-18	12	R Thalamus (Medial Dorsal Nucleus)
3	520	0.010945841	-20	-32	-20	L Culmen (Anterior Lobe)
4	488	0.013181603	-20	-6	-18	L Parahippocampal Gyrus (Amygdala)
5	416	0.011575607	42	-2	14	R Insula (BA 13)
6	392	0.011886669	-10	-46	-20	L Culmen (Anterior Lobe)

ALE, Activation likelihood estimation; R right; L, left; BA, Brodmann area.

#### 3.1.6. ALE: positive correlation with facial trustworthiness

**Fig 4:**
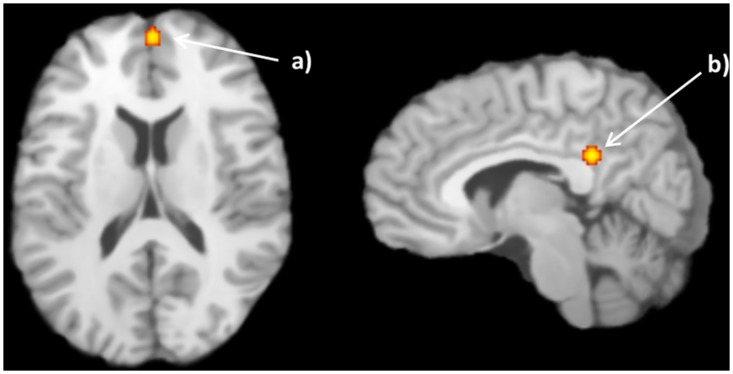
Activation likelihood estimation (ALE) meta-analysis with 6 studies (6 articles) regarding the positive correlation between neural activation and facial trustworthiness. Some of the modulated regions were a) R Cingulate gyrus (6, -43, 25) and b) R Anterior Medial Frontal gyrus (3, 55, 14). The obtained ALE maps were thresholded using 1000 permutations, *p* < .001 as cluster-forming threshold and *p* < .05 for cluster-level inference.

As to the second analysis, two clusters were found for the positive relation between faces and trustworthiness (trustworthy > untrustworthy faces), namely the medial frontal gyrus, and posterior cingulate (see Fig 4 and Table 5).

**Table 5:** ALE: results for the positive correlation. Activation likelihood estimation (ALE) meta-analysis results of regions showing a positive impact of faces trustworthiness.

Cluster #	Volume (mm^3^)	Peak ALE value	Peak coordinates			Label
			x	y	z	
1	256	0.008435635	8	-44	25	R Posterior Cingulate (BA 23)
2	232	0.009089806	2	54	16	R Medial Frontal Gyrus (BA 9)

ALE, Activation likelihood estimation; R right; L, left; BA, Brodmann area.

### 3.2. Non-quantitative analysis

Given that not all articles were eligible to be included in the quantitative meta-analyses (MA and ALE), screening of the studies/articles also not included in each quantitative analysis was nevertheless performed in order to respond to questions addressed in the systematic review.

#### 3.2.1. How does the amygdala respond to the polarity of trustworthiness signals in faces?

Considering the non-quantitative analysis, overall the studies point to an increased response of the amygdala to untrustworthy compared to trustworthy faces, showing a quasilinear profile [25, 28–31, 35, 55, 56, 58, 59], with only a few pointing to a quadratic model best fitting the amygdala response [22, 26, 32, 38] with amygdala responses both to untrustworthy and trustworthy faces. Some of these studies find evidence of both linear and quadratic responses in the right and left amygdalae [26, 31, 32] (see Table 2).

Importantly, increased responses to untrustworthy faces are found more consistently in the right amygdala, either against trustworthy faces [3, 25, 28, 30, 55–59], or against baseline periods, neutral or average-trustworthiness faces [31, 32, 37, 38]. Of the 20 included articles, whereas 9 studies found significant responses in the right amygdala specifically for the contrast untrustworthy > trustworthy faces, only 4 found the same response pattern in the left amygdala [3, 25, 31, 56] (in one study the results did not reach statistical difference after multiple correction comparison [56], but other studies reported uncorrected results, e.g. [31] (see S6 Table for a summary of results for right and left amygdalae), which favor the hypothesis that amygdala response might be lateralized during processing of trustworthiness signals [24], with stronger modulation for untrustworthiness signals. Accordingly, Pinkham et al [28] report significant differences in the right amygdala but not for the left one in the HC group. Interestingly, whereas marginal differences between untrustworthy and trustworthy faces during pre-learning phases (previously to association of faces and trustworthiness behaviors) are found in the right amygdala, the left parahippocampal gyrus/amygdala responds more to faces associated in the context of a related behavioral pattern than faces presented without such context [30]. The lateralization issue could be a potential factor explaining differences in results from studies which use faces reflecting trustworthy behaviors (e.g. [22, 35]) compared to faces rated subjectively as more trustworthy (e.g. [32, 56]). Nevertheless, Gordon and Platek [35] report that faces belonging to people which more often are engaged in trustworthy behaviors elicit both right and left amygdala activation (ROI-analysis; uncorrected data). Interestingly, this is the only study reporting increased responses to trustworthy as compared to untrustworthy faces [35]. Finally, in our systematic review and meta-analysis, although the number of studies showing significant (corrected or uncorrected) right amygdala activation for the contrast untrustworthy > trustworthy faces (n = 9) [3, 25, 28, 30, 31, 56–59] was larger than for the left one (n = 4) [3, 25, 31, 56], when directly tested the difference was not statistically significant (χ^2^(1) = 1,923, *p* = .267).

#### 3.2.2. Other regions responding to the polarity of trustworthiness signals in faces

Regarding regions besides the amygdala also involved during social cognition taken from studies which were not included in the ALE analysis, the results show some variability from study to study. Mostly, the regions which are more often reported are the insula [29, 38, 55], the cingulate cortex or adjacent areas [29, 35, 55], the superior temporal sulcus (STS) [26, 28, 55], the inferior frontal gyrus (IFG) [29, 35], the medial prefrontal cortex (mPFC) [28–30], the FG [26, 28, 29], and nuclei of the basal ganglia [29, 31, 35, 56, 57]. Of these, the pattern of responses is either linear [28, 30, 31, 35, 56, 57] or can be fitted using a quadratic model responding to both trustworthy and untrustworthy faces [26, 29, 35, 38].

The right insula is found to show increased responses to both trustworthy and untrustworthy faces compared with baseline [38] matching its left counterpart [29], although the left insula also shows a linear pattern responding more to untrustworthy than to trustworthy faces as the left anterior cingulate [39, 55]. Nevertheless, responses of right insula specifically to linear increases of facial untrustworthiness perception are also reported [36, 39]. The right cingulate shows a quadratic effect regarding trustworthiness ratings [29] with the paracingulate showing the same effect [35], and the left anterior cingulate showing linear responses to untrustworthy compared to trustworthy faces [39]. The left lateralized basal ganglia activity pattern points to a quadratic model, with the left putamen showing increased responses to both extremes of Trusting behavior [35], although linear responses to untrustworthy faces are also found [56]. The left caudate shows the same quadratic response to trustworthiness ratings of faces [26]. In contrast, the right basal ganglia seem to more often show linear responses, with the right putamen responding more to low trust faces [36, 57] and the right caudate responding in a linear positive manner to trustworthiness ratings.

As for regions particularly involved in the face network, the right STS either shows increased responses to untrustworthy faces [28] or follows a quadratic model [26]. The response of the FG is reported to best fit a quadratic model [26, 29], with the left responding more to trustworthy faces compared to baseline and the right more to untrustworthy than to baseline [29]. These results are not contrary to findings that both the left and the right FG respond more to untrustworthy faces than to trustworthy ones [28]. The activity of the IFG presents differences depending on the hemisphere: the left seems to show a linear pattern of response regarding trusting behavior [35], whereas the right one shows increased activity to both trustworthy and untrustworthy rated faces [29]. The mPFC shows increased responses to untrustworthy faces [28] although reports of quadratic effects are also found [29]. Three areas showing increased responses to trustworthy faces are the right temporoparietal junction [30], the left FG [29] and the left precuneus [39].

### 3.3. Risk of bias

#### 3.3.1 Graphical evaluation of publication bias: funnel plots

**Fig 5:**
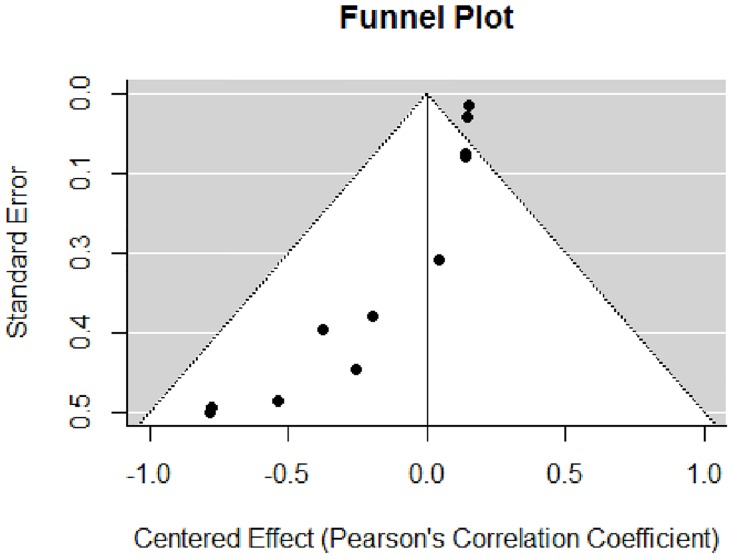
Funnel plot. Verification of publication bias in the meta-analysis of effect sizes is graphically represented in a Funnel plot displaying effect size and standard error.

The funnel plot testing publication bias within the MA is presented in Fig 5. The graphical results point to asymmetry, with a majority of the smaller studies clustering to the left of the mean.

#### 3.3.2 Algebraic evaluation of publication bias: Egger’s regression test

**Fig 6:**
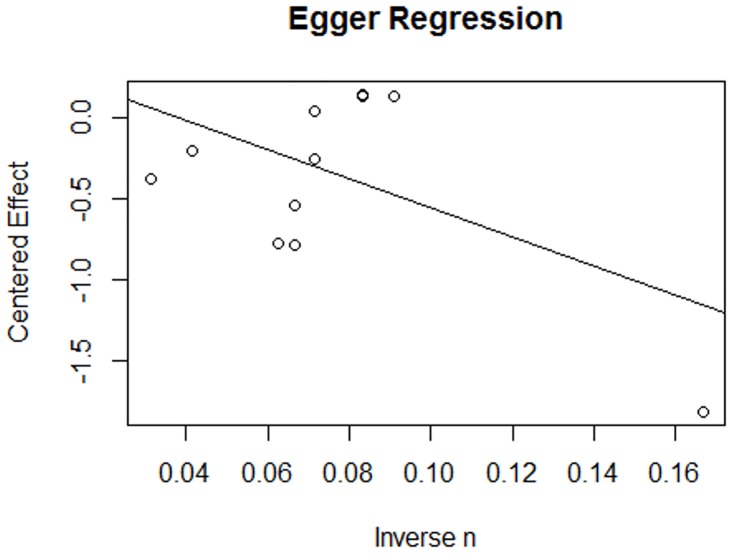
Egger’s regression. Graphical results of the regression performed to evaluate asymmetry in the results and publication bias in the meta-analysis of effect sizes.

Although the funnel plot pointed to asymmetry, Egger’s regression test revealed non-significant findings (F(1,10) = 3,63; *p* = .086), which means that asymmetry cannot be assumed for the studies included in the MA. The reported variability in the effects of the different studies is explained in 19.3% by the measured precision (inverse of the studies dimension, 1/n) (Fig 6).

## 4. Discussion

This systematic review and meta-analyses show evidence for a role of the amygdala in trustworthiness processing. Importantly, we found evidence for right lateralization, in particular in what concerns larger activation for untrustworthy compared to trustworthy faces. This evidence came both from two different sorts of analyses: MA and ALE. Also, other areas such as the posterior cingulate and medial frontal gyrus seem to be implicated in the network that processes trustworthiness signals in faces, given by the ALE analysis.

Subgroup analyses pointed to particular strong positive effects (untrustworthy > trustworthy faces) in the right amygdala, with narrower confidence intervals in studies which employed methods such as use of both explicit and implicit tasks in the paradigm, two or more categories of trustworthiness values, and spatial smoothing of fMRI data using an 8 mm kernel size. In addition, our revision of studies pointed to a higher amount of ROI-based /small volume corrected analyses compared to whole-brain ones, with results being reported with uncorrected p-values given the assumption and *a priori* evidence of amygdala involvement in these processes (e.g. [24]). Nevertheless, no significant differences in effect sizes were found between studies employing restricted volumes or whole-brain analysis.

### 4.1. How does the amygdala respond to the polarity of trustworthiness signals in faces?

#### 4.1.1. Contrast ‘untrustworthy > trustworthy’ faces

Our work systematizes and generalizes the notion that the amygdala shows larger responses for untrustworthy faces, with a right lateralization pattern. This was a clear outcome of our meta-analysis of effects that was also confirmed by ALE.

The MA pointed to evidence of increased right amygdala response to untrustworthy faces compared to trustworthy ones. Notwithstanding is the extent of the confidence interval (values between 42 and 97%), indicating that there exists a large amount of heterogeneity between studies, also due to the small sample size. Therefore, the global effect should be interpreted carefully. Ideally, the meta-analysis should be replicated with a larger sample size. Nevertheless, random effects measures allow that the results might be generalized to the population, as it considers both within- and between-study variability, even when resulting in broader confidence intervals compared to a fixed-effects analysis [34]. The MA indicated a positive effect in the right amygdala response to untrustworthy faces when compared to trustworthy ones, namely in studies that used 8 mm spatial smoothing, or studies which have used explicit and implicit experimental task paradigm or used two or three categories for the experimental paradigm instead of a Likert scale (using a continuum of values). Adding to this result, the amygdala appeared as expected as a relevant cluster in the ALE analysis. Regarding the negative correlation between faces and trustworthiness, ALE results reported clusters containing right and left amygdala among others, with the right amygdala cluster presenting a considerably higher cluster size as compared to the left amygdala cluster size. The presence of a larger cluster does not necessarily mean that there is a greater spatial extent in activity within this region. It may alternatively indicate that there is a higher variability in the spatial overlap of included coordinates across studies in a given region [49]. Nevertheless, if this is true when comparing different regions, it becomes less likely when comparing similar regions such as the right and left amygdala. There is in principle no reason to expect that similar regions would yield different spatial variability. Thus, and since the right amygdala cluster is not only bigger, but also presents higher peak values than the left one, we can conclude that there is stronger involvement of that region.

The amygdala was suggested to be involved in the extraction of trustworthiness signals from faces (e.g., [11, 15, 24, 25]) and its activity evoked by untrustworthy-looking faces had been suggested to be higher than for trustworthy-looking ones [7]. The current analyses generalize the findings that amygdala responses to faces increases with the decrease of their perceived trustworthiness, even when subjects are performing tasks that do not require explicit evaluation of faces [3, 30, 32, 56].

Moreover, studies with clinical populations show that the response of the right amygdala is diminished in clinical groups such as autism, schizophrenia and Klinefelter syndrome [15, 27–29]. Importantly, these effects seem to depend on the explicit (trustworthiness judgments) or implicit (age / gender judgments) nature of task. Baas et al. (2008) showed overall decreases in right amygdala activity during judgements of both trustworthy and untrustworthy faces for the schizophrenia (SCZ) clinical group. In the left amygdala, decreased activity was found particularly when performing judgments of trustworthy faces compared to HCs.

Interestingly, a recent structural study showed that increased right amygdala volumes are correlated with higher tendency to rate faces as both more trustworthy and untrustworthy [60] although this does not clarify if the amygdala then responds also more to facial extremes of trustworthiness.

#### 4.1.2. Linear / nonlinear response

Although this systematic review included articles showing both linear and nonlinear (quadratic) effects of facial trustworthiness in amygdala response, the studies included in the quantitative meta-analyses (MA and ALE) reported linear effects only.

From the 20 articles selected for the systematic review, 5 did however report nonlinear right amygdala responses (see Table 2). In one of these 5 articles, Freeman and colleagues suggested that the design of the task (blocked versus event-related) could influence the amygdala response [32]. They performed 2 studies. Experiment 1 results revealed coexisting linear and nonlinear responses, being suggested that the repeated presentations in the blocked-design have induced a task context that increased the tracking of valence over salience. Alternatively, Experiment 2, using an event-related design, showed evidence only of nonlinear effects. The authors referred that the event-related design of this experiment used a wider, continuous range of trustworthiness, leading to the increase of sensitivity to nonlinear effects [32, 61].

In fact, in 2 of the articles reporting nonlinear responses included in the systematic review, the amygdala seemed to behave in a similar manner, i.e., according to the design of the task. Mattavelli et al. [26] performed a task in which blocked-design was used and, as previously reported in Experiment 1 of Freeman et al. [32], the amygdala revealed both linear and quadratic responses [26, 38] (note that Mattavelli et al., [26] combined right and left amygdalae responses as they state that both hemispheres showed similar response patterns). Another article, in which the task was performed using an event-related design [22], reported only a nonlinear (right) amygdala response pattern, consistent with the findings of Freeman’s Experiment 2. However, the same behavior was not reproduced in the 2 remaining articles presenting nonlinear right amygdala responses. Despite performing a task with an event-related design, Said and colleagues reported both linear and quadratic responses [31]. Also, another study, which task was performed with a block-design, have only revealed the existence of a nonlinear representation. In fact, a direct linear contrast between untrustworthy and trustworthy faces resulted in null findings, with linear contrast results arising only between extremes values of trustworthiness and neutral faces [38].

A recent systematic review suggests another approach and states that there is compatibility between linear and nonlinear models. It is possible that these analyses are related to distinct processes, in which areas displaying linear patterns may be related to face valence, while regions presenting quadratic patterns may be associated, for example, to face intensity [16]. According to a meta-analysis that compared nonlinear against linear amygdala responses, the ventral portion of the amygdala was more responsive to negative linear contrasts, while a dorsal portion of the amygdala was more consistently active in nonlinear contrasts [16]. These results are consistent with other findings, suggesting the involvement of the ventral portion of the amygdala (linear response) in processing valence, while the dorsal portion of the amygdala (nonlinear response) would be recruited when determining the value of ambiguous information [62].

Thus, it is still not clear if event-related designs influence the amygdala to respond only in a nonlinear manner, and if blocked designs lead to the detection of both linear and nonlinear responses. Nevertheless, the data analyzed in this review (systematized in S7 Table) does not support such hypotheses.

In conclusion, it would be interesting that future studies could clarify how does the design of the task influences the type of the amygdala response and if different parts of the amygdala are involved in differential signaling of trustworthiness in faces.

### 4.2. Identification of novel areas involved in face trustworthiness processing

For the negative correlation between faces and trustworthiness, the ALE analysis revealed clusters including, among others, the amygdala and the insula, whereas for the positive correlation, areas such as the posterior cingulate and medial frontal gyrus were identified. ALE uses a random-effects model that searches spatial coherence across studies and minimizes the effect of agreement within studies [48], allowing to generalize the effects to the population. Taking also into consideration the limitations of the ALE model [43], this is a relevant and novel finding. In fact, areas such as the medial frontal gyrus and the posterior cingulate presented increased activity during social and emotional processing. Accordingly, the medial frontal gyrus has been found to be involved in personal moral judgments, and likewise, the posterior cingulate cortex, also implicated in personal moral judgments, reveals increased neural activity for familiar faces and voices [63]. Additionally, for the negative correlation between faces and trustworthiness, a cluster in the right insula appeared, reflecting the impact of face untrustworthiness in neural responses. Previous work shows that the insula is involved in the perception and representation of emotional and affective states, playing an important role in the network underlying social decisions [15]. This result is also consistent with previously reported studies in the literature, since the insula was considered a critical region when performing trustworthiness judgments by responding to low levels of trust, in particular for untrustworthy faces, whether or not trustworthiness was being explicitly assessed [64, 65]. A recent study has found that the tendency to trust is positively reflected in the volumes of structures like the bilateral ventromedial prefrontal cortex (vmPFC) and bilateral anterior insula [60]. Therefore, considering the results of this ALE analysis, one can infer that these regions belong to the network that processes trustworthiness signals in faces, although one should be careful in the establishment of direct correlations between functional and structural data.

### 4.3. Factors affecting the study of trustworthiness

Importantly, although a thorough list factors have been extracted from the original articles and considered to form subgroups, only 3 showed to be relevant for positive effects in the MA, namely the task performed, the trustworthiness type of categorization, and the smoothing applied to data.

In a previous analysis of studies in the literature, Morawetz et al. [66] showed that size of the amygdala activation increased, as expected, depending on the option of spatial filtering options used: none, 4 mm or 8 mm. The size of 8 mm for the smoothing kernel increased on average five times the activation volume seen in amygdala, compared to the use of no filter [66]. The authors conclude that excessive spatial smoothing should be omitted to preserve regional specificity and sensitivity. Our subgroup analysis showed a positive effect (untrustworthy > trustworthy faces) in studies using a kernel of 8 mm [25, 32, 55], but not in studies using smaller kernel sizes (4, 6 and 7 mm), which is intriguing. In fact, Bos et al. [55] report null effects which suggests that the likelihood of increased effects does not necessarily hold.

Studies using a mix of explicit and implicit tasks in the fMRI paradigm [25, 57] also present a more clear effect of the contrast, compared to studies only employing implicit tasks or employing both implicit and explicit ones but analyzing only the later one (see S1 and S4 Tables). The type of task has been shown to differentially recruit the amygdala depending if the task requires an implicit or an explicit emotional label [67–69], with meta-analyses findings either pointing to increased amygdala responses to explicit tasks [70] or no differences between explicit label of facial emotion and attended incidental processing of stimuli. Passive viewing showed the best odds of activation [71]. Moreover, the nature of task does not seem to affect laterality of amygdala activation [72].

Concerning trustworthiness categorizations, studies using strict categorical conditions [55, 58] show a more reliable positive effect than studies employing a continuum of trustworthiness values [3, 25, 30–32, 35, 56, 57, 59]. In fact, some variability in the methodology used concerning categorization of trustworthiness values is found between studies (see S8 Table). Whereas in some of the studies trustworthiness categorizations into “trustworthy” or “untrustworthy” rely on judgements performed by the participants that also perform the main task (in explicit tasks the judgements are part of the main study), others rely on judgements made by different participants. This would potentially introduce a bias, as trustworthiness judgements are subjective. Nevertheless, the amygdala seems to respond more consistently to consensus ratings of trustworthiness than to idiosyncratic ones [3, 22], indicating that some features are recognized as trustworthy and as untrustworthy in the general population. Importantly, in one of the articles [39], the assignment of faces to the trustworthiness conditions was arbitrary and counterbalanced across participants. This might explain why the direct contrast of untrustworthy versus trustworthy faces did not yield significant results in the amygdala region.

Although differences in methodology of analysis such as the use of regions of interest or whole-brain analysis do not seem to show differences in terms of global effects in amygdala response to facial trustworthiness, findings resulting from these studies might nevertheless be emphasized. In fact, ROI-based and small volume correction methods imply reduction of voxels for correction of multiple comparisons. Many of the studies collected in this systematic review performed ROI-based analysis / small volume correction and reported also uncorrected results, given the *a priori* hypothesis related with the amygdala involvement in social cognition, and in particular, trustworthiness judgements. This hypothesis is based on seminal lesion studies [24], being corroborated by studies performed in clinical populations (e.g. autism, schizophrenia) in which the function of the bilateral amygdala is thought to be corrupted, leading to the decrease of the amygdala response (and also of other structures) to untrustworthy faces as compared with HCs (e.g. [15, 27–29]. Therefore, more stringent criteria are required in future studies, for instance, the use of whole-brain analyses with correction for multiple comparisons, with ROI-based / small volume correction analyses being used as a complementary method to ask more specific questions within that region.

Moreover, differences between studies addressing trustworthiness based on facial judgements and based on associations of faces and behavioral patterns throughout the task should be taken in consideration, as different aspects of trustworthiness processing are being analyzed, namely perception versus learning. Importantly, first impressions should not be disregarded as there is evidence that amygdala activation reflects more directly impressions of trustworthiness than the actual trustworthiness [22]. This might explain amygdala responses to untrustworthy faces during pre-learning phases of trustworthy behaviors [30].

### 4.4. Trustworthiness evaluation using other brain function assessment techniques

To our knowledge, almost all studies evaluating the neuronal processes underlying facial trustworthiness are based on fMRI measures. However, other studies have been performed using other methods, like event-related brain potentials (ERP) through the use of Electroencephalography (EEG), which have the advantage of higher temporal resolution.

A study evaluating how facial trustworthiness affected facial processing have shown that trustworthy faces elicited a more positive C1 (earliest evoked visual component peaking negatively between 50–90 ms after stimulus onset) than untrustworthy faces. The authors suggest that since C1 was modulated by face-type, the discrimination between trustworthy and untrustworthy faces was performed in this early stage of visual processing. Also, untrustworthy faces elicited a more positive late component (LPC) than trustworthy faces, suggesting that a greater amount of processing related to feedback signaling was allocated to faces categorized as untrustworthy [73].

Additionally, a study that investigated the temporal dynamics of trustworthiness perception revealed that explicit trustworthiness judgments elicit an enhanced early posterior negativity (EPN), with an amplitude enhancement for untrustworthy male faces and trustworthy female faces. The authors speculate that the negativity in the ERP during trustworthiness judgments accompanies the relevance of the faces that should be remembered in future social interactions [74]. The negativity recorded during these judgments was interpreted as reflecting a higher depth of processing relevant faces. According to their suggestion, this could result from amygdala back projections to the cortex, thus reinforcing the coding of these faces for more effective future interactions [74].

Along with the EPN, a right lateralized effect was also demonstrated, in line with other studies that revealed a primary role of the right hemisphere in face emotional recognition, in particular for stimuli with negative valence [74, 75]. In fact, the meta-analysis performed in our study has also shown that the right amygdala in particular revealed higher responses for stimuli presenting negative valence (in this case, for untrustworthy faces).

### 4.5. Risk of bias and limitations

Our systematic review applied some methods in order to minimize the introduction of bias in the literature search and results. First, the literature search was performed without using “amygdala” as one of the keywords. In fact, although there is primary evidence mainly from lesion studies that the amygdala is involved in extraction of information during trustworthiness judgments [24, 76], we were interested in evaluating the role of the amygdala within a large set of areas which are also implied in trustworthiness processing. Second, our inclusion criteria considered for ALE only whole brain studies (excluding ROI-based ones which define *a priori* specific regions). Third, a fully unbiased analysis was performed by considering all the results (irrespective of significance and null effects) found in the literature, both for the MA and the ALE. One point must be made however, stating an important distinction between ALE and meta-analyses of effect sizes. In fact, whereas a null-effect is relevant within a meta-analysis of effect sizes, as the later assesses the pooled strength of an effect, ALE measures are only concerned with probabilistic location sites, and therefore null-findings do not influence ALE results. This is an important point within risk of bias and limitations of this method as it emphasizes the existence of a given area while disregarding the number of studies in which that area did not appear. Forth, measures of consistency and heterogeneity (I^2^, Q) were employed in the MA to explore variability between studies. Heterogeneity was found either using Cochran Q and I^2^ results, however, whereas Q is sensitive to the number of included studies [77], which might be a limitation as we used 12 studies in the MA, I^2^ does not rely on this measure to predict heterogeneity, relying instead on the sample sizes within studies [78]. Heterogeneity in our MA might then have arisen from studies with smaller sample sizes.

Moreover, regarding reporting bias, we are aware that including ROI-based / small volume correction studies in the meta-analyses of effect sizes, and by including uncorrected results in the ALE analysis, our results regarding the amygdala and other regions might even so be emphasized. But, we should point that studies using ROI-based analysis or small volume correction studies did not restrict these analyses to the amygdala, as ROIs / small volume correction were also used in regions implicated in social perception and cognition [79] such as the FG [26, 28, 29, 32], STS [26, 28, 29, 37], temporal pole [55], insula [29, 36, 38, 55, 58], anterior cingulate cortex [55], orbitofrontal cortex [57, 58], mPFC [28], and ventral lateral prefrontal cortex [28]. Nevertheless, *a priori* hypothesis justifying ROI analysis / small volume correction were more often devoted specifically to the amygdala region [22, 30–32, 35, 37, 55, 56], which suggests a dominant preference for the amygdala in studies addressing trustworthiness.

One might point that 11 articles in the MA, or 6 articles in the ALE might limit power for more comprehensive statistical inference. although meta-analysis with only 3 articles [80] are not rare. In this respect, Yaffe et al [81] have made a consideration regarding empty reviews [81, 82] as they usually (1) offer no conclusions, (2) offer conclusions based on referenced excluded studies, (3) offer conclusions based on other evidence, or (4) offer conclusions not based on evidence [81]. These reviews are still informative in the sense they detail reasons for exclusion adding cues regarding lack of data or possible flaws in the research field. Reasons for empty reviews, which mimic reasons for few studies reviews, are (a) very recent areas of studies; (b) ask research questions which are very specific; or (c) the inclusion criteria are methodologically very demanding in the sake of quality evidence [81]. As far as we know, the amygdala role in social cognition, and in trustworthiness processing, is largely addressed in the literature (a search in PubMed using “amygdala AND trustworth*” returned 40 articles, whereas using “amygdala AND (social cognition)” returned 505) and the questions posed in this systematic review are addressed by at least 20 articles. Our systematic review and the small number of studies which were finally included in the meta-analysis can be nevertheless explained by the reason (c), the criteria were methodologically demanding as we decided to include only papers directly comparing conditions of trustworthy and untrustworthy faces, respecting lateralization of amygdala activation (only right amygdala results were considered for the meta-analysis of effect sizes) or which referred to whole-brain analysis (ALE). In this manner, it was our goal to minimize bias in the results of this systematic review.

Finally, in order to evaluate publication bias in the meta-analysis of effect sizes, both funnel plots and Egger’s regression test were performed. Although the funnel plot shows a trend for asymmetry, the Egger’s test did not find conclusive evidence for such bias.

## 5. Conclusions

These systematic review and meta-analyses provide an overview of neuroimaging studies regarding the cognitive neuroscience of facial trustworthiness processing. We found evidence for an important role of the amygdala in the social network involved in facial trustworthiness processing, particularly in which concerns untrustworthy faces, despite high heterogeneity between studies. Activation likelihood estimation (ALE) was consistent with these findings and highlighted an important role for both the amygdala and insula, since these are two of the most commonly involved brain regions when evaluating others’ trustworthiness from faces.

We also found evidence for novel regions involved in trustworthiness processing, namely the posterior cingulate and medial frontal gyrus. Future studies should aim to elucidate the role of these regions in affective processing of trust in health and disease.

Importantly, the heterogeneity found between studies suggests that little consistency exists in the methodology of study design/data acquisition/analysis in the trustworthiness literature. Therefore, particular attention to this issue should be paid, and more stringent criteria should also be used in fMRI analyses given the risk of bias whenever a particular *a priori* hypothesis exists.

## Supporting Information

S1 File PRISMA checklist. (DOC)

S1 Fig Forest plot. Forest plot displaying results of the subgroup analysis.
(TIFF)

S1 Table Characterization of the articles (n = 20) included for systematic review. (A) experimental design, paradigm and stimuli; (B) population, acquisition and analysis parameters.
(PDF)

S2 Table Inclusion or exclusion criteria for MA and ALE. Meta-analyses and ALE: decision of inclusion or exclusion of the articles and studies.
(PDF)

S3 Table Meta-analysis of effect sizes: characterization of studies and data. Meta-analysis of effect sizes: population characterization, original values (t-scores and Z-scores), contrasts, type of analysis, p-values and corrections taken from the studies feasible for meta-analysis for the contrast "Untrustworthy > Trustworthy" or correlation with facial trustworthiness scores in the (right) amygdala.
(PDF)

S4 Table Subgroups analysis. Subgroups analysis: division into subgroups generated according to methodological components taken from the experimental design, data acquisition and analysis parameters.
(PDF)

S5 Table ALE: characterization of studies and data. (A) Articles selection for the negative correlation between faces and trustworthiness (Untrustworthy > Trustworthy); (B) Articles selection for the positive correlation between faces and trustworthiness (Trustworthy > Untrustworthy).
(PDF)

S6 Table Lateralization of amygdala. Lateralization of amygdala activation within the 20 articles included in the systematic review.
(PDF)

S7 Table Amygdala activation, study design and linearity. Studies displaying results in amygdala, organized according to ventral-to-dorsal activation (considering X coordinates), study design and linearity (linear, non-linear/quadratic) of response.
(PDF)

S8 Table Facial trustworthiness judgements. Categorization of facial trustworthiness judgements.
(PDF)
