# Supplementary figures and images for: The Role of the Amygdala in Facial Trustworthiness Processing: A Systematic Review and Meta-Analyses of fMRI Studies
Source: PLoS One. 2016 Nov 29;11(11):e0167276. doi: 10.1371/journal.pone.0167276 (PMC5127572; doi:10.1371/journal.pone.0167276)

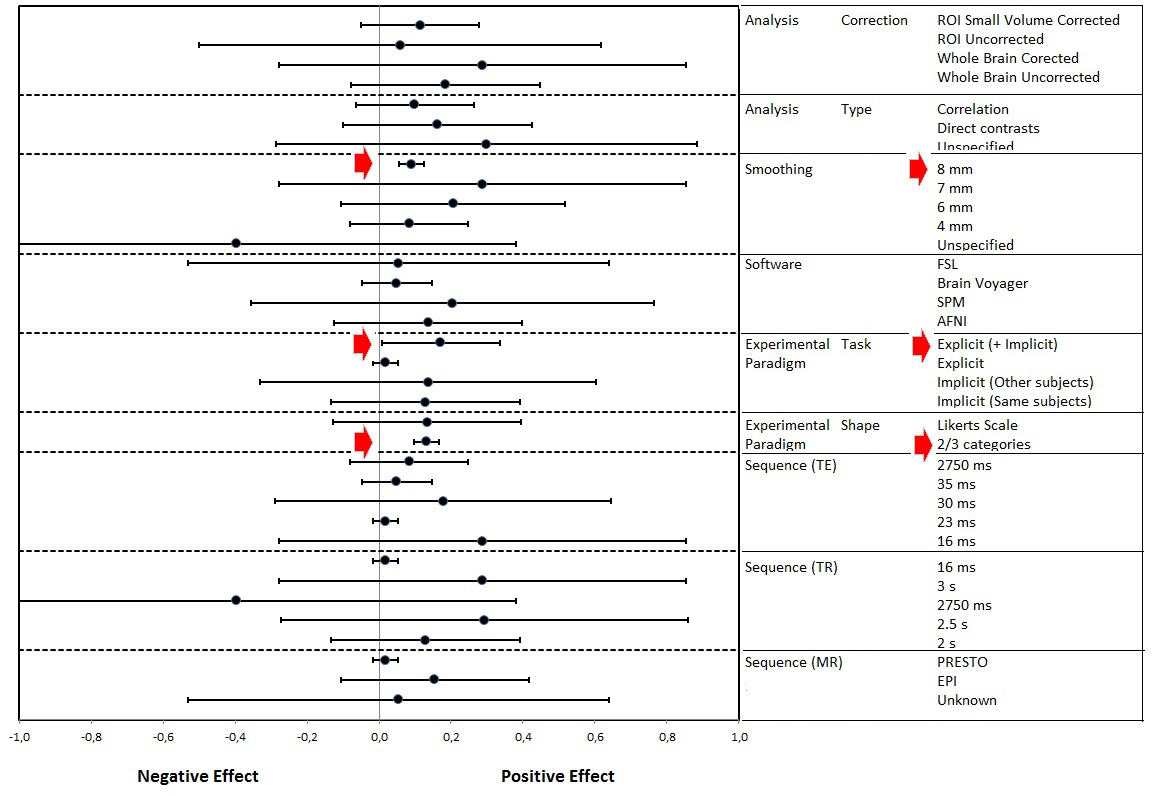

Supplement: S1 Fig — Forest plot displaying results of the subgroup analysis. (TIFF) [file pone.0167276.s002.TIFF]
