# Supplementary material for: The Role of the Amygdala in Facial Trustworthiness Processing: A Systematic Review and Meta-Analyses of fMRI Studies
Source: PLoS One. 2016 Nov 29;11(11):e0167276. doi: 10.1371/journal.pone.0167276 (PMC5127572; doi:10.1371/journal.pone.0167276)
Supplement: S1 Table — (A) experimental design, paradigm and stimuli; (B) population, acquisition and analysis parameters. (PDF) [file pone.0167276.s003.pdf]

S1 Table. Characterization of the articles (n=20) included for systematic review. (A) population, acquisition and analysis parameters; (B) experimental design, paradigm and stimuli.

A

#	Article	Population	Sample size	Gender (M, F)	Age range (mean±SD)	System (Tesla)	Sequence	Analysis (software)	Template	Smoothing
1	Baron et al., 2011	Healthy controls	24	15M, 9F	19-29 (23)	3T Siemens Allegra	EPI (TR=2000 ms, TE=30 ms, flip angle=80º, matrix size=64x64); near whole brain coverage: 33 interleaved 3-mm axial slices	Analysis of Functional Neuro-images software (AFNI; Cox, 1996)	(Talairach, MNI)	6 mm
2	Bos et al., 2012	Healthy controls (placebo-controlled testosterone administration study)	16	16F	- (20.8±2.0)	3T Philips Achieva MRI scanner	3D PRESTO (T2*-weighted sagittal whole-brain images; TE=23 ms, TR=16 ms; FOV=224×224×136 mm; flip angle =9°); 2 runs of 450 scans with a volume acquisition time of 0.813 s, each volume: 39 sagittal slices; voxel size 3.5 mm isotropic.	SPM5	Talairach space (Talairach and Tournoux, 1998)	8 mm
3	Doallo et al., 2012	Healthy controls	12	4M, 8F	20-31 (-)	3T Tim Trio Siemens scanner	EPI (T2*-weighted; E=30 ms, TR=3 s, flip angle=87º, matrix=64x64; FOV=192 mm; voxel size=3x3x3 mm3). 45 contiguous transversal slices covered the whole brain	SPM5	MNI	7 mm
4	Engell et al., 2007	Healthy controls	15	9M, 5F	- (22.4)	3T Siemens Allegra	EPI, (TR = 2000 msec, TE = 30 msec, flip angle = 90º, matrix size = 64 x 64). Near whole-brain coverage: 33 interleaved 3-mm axial slices	AFNI	MNI	6 mm

5	Freeman et al., 2014	Healthy controls	15 (exp. 2)	4M, 12F (1 was later excluded) (exp. 2)	18-35 (21.80) (exp. 2)	3T Philips Intera Achieva Scanner	EPI (TR=2000 ms, TE=35 ms); 35 interleaved oblique-axial slices (3x3x4mm3 voxels; no slice gap) parallel to the AC-PC line	BrainVoyagerQX (Brain Innovation)	Talairach space (Talairach and Tournoux, 1998)	6 mm
6	Gordon et al., 2009	Healthy controls	6	4M, 2F	21-29 (23.7)	3T Siemens Magnetron Trio Scanner	TR=2750ms; TE=30ms; Flip Angle=90; FoV=192; Slice Thickness=2mm & 0.8mm gap; images were collected at a 30º angle to the AC-PC line to minimize distortion of ventral PFC and amygdala	FSL package of programs (Smith et al, 2004).	Talairach	-
7	Killgore et al., 2013	Healthy controls	39	22M, 17F	18-45 (29.9±8.6)	3T Tim Trio Siemens scanner	T2∗-weighted (TR=3.0 s,TE=30 ms, flip angle=90º FoV=22.4; matrix: 64 × 64 acquisition); 43 transverse slices (3.5 mm thickness, 0 skip) using an interleaved sequence, 80 images collected per slice	SPM8	MNI	6 mm (resliced to 2×2×2 mm voxels)
8	Kim et al., 2012	Healthy controls	12	12M	- (23.4±2.13) (3 were later excluded)	3T Forte MRI scanner (ISOL Technology)	EPI (T2*-weighted EPIs with BOLD contrast; 64 × 64 voxels, TR=2000 msec, TE=30 msec, flip angle = 80°, 3×3 mm in-plane resolution, slice thickness = 4 mm, 24 oblique axial slices with no gap)	SPM2	MNI	6 mm
9	Kragel et al., 2014	Healthy controls (sample of adolescent females)	43	43F	10-20 (14.7)	3T General Electric MR 750 system	Sensitivity-encoded (SENSE) spiral-in pulse sequence along the axial plane (TR=2000 ms; TE=30 ms; matrix=64x128; alfa=70º; voxel size=3.8x3.8x3.8mm3; 34 contiguous slices).	SPM8	MNI	4 mm

10	Mattavelli et al., 2012	Healthy controls	20	10M, 10F	18-35	3T HD MRI system with an eight channels phased array head coil	EPI (T2*-weighted with BOLD contrast; TR=3 s, TE=32.7 ms, flip-angle=90º, acquisition matrix 128x128, FOV=288x288 mm); whole head volumes: 38 contiguous axial slices, in-plane resolution of 2.25x2.25 mm and a slice thickness of 3mm; slices positioned for each participant to ensure optimal imaging of temporal lobe regions (including amygdala)	FSL-FEAT (FMRI Expert Analysis Tool)	MNI	6 mm
11	Pinkham et al., 2008a	Schizophrenia schizoaffective disorder (paranoid, non-paranoid), Autism spectrum disorder, Healthy controls	12	12M	18-35 (27.08±3.99)	3T Siemens Allegra	EPI (T2*-weighted images with BOLD contrast (2D; TR=2 s, TE=30 ms, flip angle=80, 32 transverse slices, voxel size= 3.8×3.8×3.8 mm, matrix=64×64; FOV=243×243)	SPM2 and the WFU Pick Atlas (Maldjian et al., 2003)	MNI	8 mm
12	Pinkham et al., 2008b	Schizophrenia schizoaffective disorder (paranoid, non-paranoid), Healthy controls	12	12M	18-35 (27.08±3.99)	3T Siemens Allegra	EPI (T2*-weighted with BOLD contrast; 2D; 32 transverse slices, voxel size 3.8x 3.8x3.8 mm, matrix = 64 x 64; FOV=243x243, TR=2 s, TE=30 ms, flip angle = 80)	SPM2	MNI	8 mm
13	Platek et al., 2008	Healthy controls	11	4M, 7F	- (-)	3T scanner	Rapid event-related fMRI (2×300 42 slice (10% gap) full brain coverage volumes; TR = 2.5, TE = 30 (ms), FoV= 19.2 cm, flip angle = 85)	FSL-FEAT	MNI	6 mm

14	Rule et al., 2013	Healthy controls	14	14F	- (-)	3T Siemens Tim Trio Scanner	EPI (T2*-weighted with BOLD contrast; TR=2,000 ms); 31 axial slices per whole-brain volume, 3-mm in-plane resolution, 4-mm thickness,0-mm skip	SPM8	Talairach	8 mm
15	Ruz et al., 2011	Healthy controls	18	8M, 10F	20-31 (-)	3T Trio scanner	EPI (T2*-weighted; TR=2.1 s; TE=22 ms, flip angle=90°); 35 interleaved sagittal slices with a thickness of 4 mm, tilted 30º to the AC–PC line to optimize sensitivity to orbitofrontal cortex and medial temporal lobes covered the entire brain (64×64 matrix with a field of view of 192×192 mm, voxel size of 3×3×4 mm)	SPM5	MNI	8 mm3
16	Said et al., 2009	Healthy controls	32	17M, 15F	- (22.8±6.8)	3T Siemens Allegra	EPI (TR = 2000 msec, TE = 33 msec, flip angle = 908, matrix size, 64x64); whole brain coverage was achieved with 34 interleaved 3.6-mm axial slices with an interslice gap of 0.36 mm	AFNI, Analysis of Functional NeuroImages (Cox, 1996)	MNI	4-mm
17	Todorov et al., 2008	Healthy controls	14	7M, 7F	18-27 (-)	3T Siemens Allegra	EPI (TR=2000 ms, TE¼30 ms, flip angle=80º, matrix Size=64x64). By using 33 interleaved 3-mm axial slices; near whole brain coverage	AFNI, Analysis of Functional NeuroImages (Cox, 1996)	Talairach	6mm
18	Tsukiura et al., 2013	Healthy controls	23	23F	- (23.8±2.2)	3T Philips Achieva scanner	EPI (BOLD contrast; 64x64 matrix, TR=2000ms, TE=30 ms, flip angle=70º, FOV=24 cm, 34 slices, 3.75mm slice thickness)	SPM8	MNI	8-mm
19	van Rijn et al., 2012	Klinefelter syndrome (KS);	18	18M	- (32.2±9.4)	1.5T Philips ACSNT	2D-EPI (BOLD contrast; TE=40 ms; TR=2500 ms;	SPM2	MNI	6mm

		Healthy controls				scanner	flip angle=90º; FoV=192x192 mm); each volume comprised 33 axial scans with 2.2mm slice thickness (and a gap of 0.8 mm); voxel size was 3mm isotropic			
20	Winston et al., 2002	Healthy controls	12	8M, 8F	18-30 (23.3)	2T Siemens VISION system	EPI (T2*-weighted images with BOLD contrast; TR=2.5 s); each volume comprised 33 × 2.2 mm axial scans with 3-mm in-plane resolution	SPM99	Talairach	8-mm

B

#	Article	Dimensions studied	**Type of task** *implicit, explicit (subliminal, supraliminal)*	**Type of stimulus** *neutral-emotional (real, avatar)*	Stimulus presentation	Paradigm	Baseline condition	Response type
1	Baron et al., 2011	Trustworthiness	Implicit (memory task – cover story)<br><br>(supraliminal)	Neutral faces (with direct eye-gaze)<br><br>(real faces)	static	Event-related	Fixation cross	Indicate whether or not they had seen the 'test-image' (Button press)
2	Bos et al., 2012	Trustworthiness	Explicit (trustworthiness judgements) and implicit (age judgements) tasks<br><br>(supraliminal)	Neutral faces<br><br>(real faces)	static	Event-related	Fixation cross	Button press
3	Doallo et al., 2012	Inhibitory control and emotional devaluation (trustworthiness)	Explicit (trustworthiness judgements) and implicit (ethnicity judgements) tasks<br><br>(supraliminal)	Neutral faces (with direct eye-gaze)<br><br>(real faces)	static	Event-related	Fixation cross	Button press (index or middle finger of the left or right hand)

4	Engell et al., 2007	Trustworthiness (idiosyncratic perception or consensus ratings)	Implicit (memory task/identity judgements)<br><br>(supraliminal)	Neutral faces (with direct eye-gaze)<br><br>(real faces)	static	Block-design	Fixation cross	Report whether the identity of the test face was the same as any of the faces in that block<br>(Button press)
5	Freeman et al., 2014	Trustworthiness	Passive viewing<br><br>(subliminal and supraliminal)	Neutral faces (with direct eye-gaze)<br><br>(real faces, exp. 2)	static	Experiment 1 (block- design); Experiment 2 (event-related)	Fixation cross	Passive viewing
6	Gordon et al., 2009	Trust behaviour, Psychopathy, Machiavellianism	Implicit (gender judgements)<br><br>(supraliminal)	Neutral faces (with direct eye-gaze)<br><br>(real faces)	static	Event-related	Fixation cross	Make sex discrimination of a face while being scanned<br>(Button press)
7	Killgore et al., 2013	Trustworthiness; Emotional intelligence	Passive viewing<br><br>(supraliminal)	Faces (neutral, trustworthy, untrustworthy)<br><br>(avatar faces)	moving	Block design	Fixation cross	Button press (dominant hand)
8	Kim et al., 2012	Trustworthiness; First impressions	Implicit (Ultimatum game)<br><br>(supraliminal)	Neutral faces (with direct eye-gaze)<br><br>(real faces)	static	Event-related	Fixation cross	Accept or reject the offer<br>(Button press)
9	Kragel et al., 2014	Trustworthiness; Age-related changes	Explicit (trustworthiness judgements)<br><br>(subliminal and supraliminal)	Neutral faces (with direct eye-gaze)<br><br>(real faces)	static	Event-related	Fixation cross	Four symbols ('--', '-', '+' and '++') prompting for a response ranging from low to high trustworthiness<br>(Button press)
10	Mattavelli et al., 2012	Trustworthiness	Explicit (trustworthiness judgements) and implicit (gender judgements) tasks<br><br>(supraliminal)	Faces (varied in pose, age and expression)<br><br>(real faces)	static	Block design	Fixation cross	Instructed to look at the stimuli and press with the right index finger a whenever they saw the red spot<br>(Button press)

11	Pinkham et al., 2008a	Trustworthiness	Explicit (trustworthiness judgements) and implicit (age judgements) tasks<br><br>(supraliminal)	faces taken from the Trustworthiness/ Approachability Task (Adolphs et al., 1998).<br><br>Neutral faces (with direct eye-gaze)<br>(real faces)	static	Event-related	Fixation cross	Button press
12	Pinkham et al., 2008b	Trustworthiness	Explicit (trustworthiness / approachability task)<br><br>(supraliminal)	Neutral faces (with direct eye-gaze) (faces taken from the Trustworthiness/ Approachability Task (Adolphs et al., 1998))<br><br>(real faces)	static	Event-related	Fixation baseline	Button press
13	Platek et al., 2008	Trustworthiness	Implicit (gender and ethnicity judgements)<br><br>(supraliminal)	Neutral faces (with direct eye-gaze)<br><br>(real faces)	static	Event-related	A jittered ISI with null events consisting of scrambled luminance matched faces	Response in the period 2000s following stimulus; participants were instructed not to respond to the null event<br>(Button press)
14	Rule et al., 2013	Trustworthiness	(study 5 – fMRI)<br>Implicit (face symmetry judgements)<br><br>(supraliminal)	Neutral faces (with direct eye-gaze)<br><br>(real faces)	static	Event-related	Fixation cross	Button press
15	Ruz et al., 2011	emotional conflict (given by trustworthiness profiles)	Explicit (Ultimatum game – the partner was signalized/cued as trustworthy or as untrustworthy)	Karolinska Directed Emotional Faces database (happy, angry)	static	Event-related	Fixation point	Accept or reject the offers (index and middle fingers of their right hand)<br>(Button press)

		during interpersonal interactions	(supraliminal)	(real faces)				
16	Said et al., 2009	Trustworthiness	Explicit (trustworthiness judgements)<br><br>(supraliminal)	Neutral faces (with direct eye-gaze)<br><br>(real faces)	static	Event-related	Fixation cross	Button press
17	Todorov et al., 2008	Trustworthiness; Valence (avoidance - approach) evaluation	Implicit (memory task/identity judgements – cover story)<br><br>(supraliminal)	Faces (neutral, trustworthy, untrustworthy)<br><br>(avatar faces)	static	Event-related	Fixation cross (12-s)	Button press
18	Tsukiura et al., 2013	Trustworthiness	Explicit (rate personality goodness on the basis of trustworthiness)<br><br>(supraliminal)	Neutral male faces (with direct eye-gaze)<br><br>(real faces)	static	Event-related	Fixation interval	Button press (8-button response box)
19	van Rijn et al., 2012	Trustworthiness	Explicit (trustworthiness judgements) and implicit (age judgements) tasks<br><br>(supraliminal)	Neutral faces (with direct eye-gaze)<br><br>(real faces)	static	Block-design	Baseline blocks (45 s)	Button press
20	Winston et al., 2002	Trustworthiness	Explicit (trustworthiness judgements) and implicit (School-University[age] judgements) tasks<br><br>(supraliminal)	Neutral faces (although screened with happy, sad, angry, disgust, fear, surprise ratings)<br><br>(real faces)	static	Event-related	Fixation cross	Button press

Legend: -, missing information; M, males, F, females; BOLD, blood oxygenation level-dependent; FOV, field of view; EPI, echo-planar functional imaging; TE, echo time; TR, repetition time.
