# Supplementary material for: The Role of the Amygdala in Facial Trustworthiness Processing: A Systematic Review and Meta-Analyses of fMRI Studies
Source: PLoS One. 2016 Nov 29;11(11):e0167276. doi: 10.1371/journal.pone.0167276 (PMC5127572; doi:10.1371/journal.pone.0167276)
Supplement: S2 Table — Meta-analyses and ALE: decision of inclusion or exclusion of the articles and studies. (PDF) [file pone.0167276.s004.pdf]

Table S2 – Meta-analyses of effect sizes and ALE: decision of inclusion or exclusion of the articles and experiments.

#	Study	ROI/ Mask/ Whole-brain	Contrasts tested	Criteria for INCLUSION / REJECTION	Decision
1	Baron et al., 2011	Whole-brain (p < .001, uncorrected)	Untrustworthy > trustworthy faces (pre-learning period)	**MA**: values uncorrected for multiple comparisons**;**<br>**ALE:** no available coordinates.	MA included<br>ALE excluded
2	Bos et al., 2012	Mask restriction: small volume correction (p<.05)	Untrustworthy > trustworthy faces (placebo condition)	**MA**: it reports non-significant results for the placebo condition;<br>**ALE:** not whole-brain, but small volume corrected instead.	MA included<br>ALE excluded
3	Doallo et al., 2012	Whole-brain (FDR corrected for multiple comparisons, p<.05)	No-Go-Low-Trust faces > No-Go-High-Trust faces	**MA:** values corrected for multiple comparisons;<br>**ALE**: it tests the contrast of interest (untrustworthy > trustworthy); whole brain analysis; values corrected for multiple comparisons;	MA included<br>ALE included
4	Engell et al., 2007	**MA**: Mask restriction: small volume correction (cluster at p<.05)<br>**ALE**: Whole-brain (cluster correction at p<.005)	Linear modulation correlated with consensus ratings (untrustworthy > trustworthy faces)	**MA**: values corrected for multiple comparisons (cluster correction);<br>**ALE**: it tests the contrast of interest; whole-brain analysis; values corrected for multiple comparisons (cluster correction).	MA included<br>ALE included
5	Freeman et al., 2014	ROI / Mask restriction: small volume correction (cluster at p<.05)	Linear relation with trustworthiness	**MA:** (experiment 2 – event-related) it reports non-significant linear modulation results (only the quadratic ones are significant);<br>**ALE:** not whole-brain, but ROI-based analysis instead.	MA included<br>ALE excluded
6	Gordon et al., 2009	**MA:** ROI (p<.005, uncorrected)<br>**ALE:** whole-brain (p<.05)	Linear model of activation for faces rated with high Trusting Behavior (not for the quadratic one)	**MA**: values uncorrected for multiple comparisons;<br>**ALE:** whole-brain, uncorrected for multiple comparisons	MA included<br>ALE included
7	Killgore et al., 2013	Mask restriction (small volume correction) at p <.001 (uncorrected), or p <.10 (FDR corrected, k≥10 contiguous voxels) for insula, ACC, amygdala and gyrus rectus	Increasing Trustworthiness > Decreasing Trustworthiness<br><br>Decreasing Trustworthiness > Increasing Trustworthiness	**MA and ALE**: although the contrast met the inclusion criteria, no regions showed significant differences (but reports amygdala/other regions in contrasts untrustworthy/trustworthy vs. neutral faces). The null results were included in ALE.	MA excluded<br>ALE included
8	Kim et al., 2012	ROI / Mask restriction: small volume correction (p<.005, uncorrected)	Negative correlation with facial trustworthiness at the time of face display	**Meta-analysis**: values uncorrected for multiple comparisons;<br>**ALE:** not whole-brain, but ROI-based analysis instead.	MA included<br>ALE excluded

9	Kragel et al., 2014	Mask restriction: small volume correction	Increase with untrustworthiness independent of age	**MA and ALE:** no contrast of interest (contrast vs. baseline, not vs. trustworthy faces);<br>**ALE**: not whole-brain, but small volume correction instead.	MA excluded<br>ALE excluded
10	Mattavelli et al., 2012	ROI	Linear regressions with trustworthiness	**MA:** it reports only values of concatenated bilateral amygdala ROIs - no specific values of R amygdala;<br>**ALE:** not whole-brain, but ROI-based analysis instead.	MA excluded<br>ALE excluded
11	Pinkham, Hopfinger, Pelphrey, Piven and Penn, 2008a	ROI	Trustworthiness judgements vs. baseline; main effects of trustworthiness; and trustworthiness vs. age	**MA and ALE:** no contrast of interest.	MA excluded<br>ALE excluded
12	Pinkham, Hopfinger, Ruparel and Penn, 2008b	ROI / small volume correction	Untrustworthy > Trustworthy	**MA:** although the contrast met the inclusion criteria, no available statistical values (t, Z ou r) were available at the time of performing the meta-analysis (only approximations to p-values); statistical values are now included in Supplementary Table S3;<br>**ALE:** not whole-brain, but ROI-based analysis instead.	MA excluded<br>ALE excluded
13	Platek et al., 2008	Whole-brain (uncorrected, $p<.005$)	Negative association with consensus ratings of trustworthiness in self2ethnic faces	**MA:** values uncorrected for multiple comparisons;<br>**ALE**: it tests the contrast of interest; whole-brain analysis; values uncorrected for multiple comparisons;	MA included<br>ALE included
14	Rule et al., 2013	**MA**: Mask restriction: small volume correction<br>**ALE**: Whole-brain	Trustworthiness: linear regressor when controlling for the quadratic regressor	**MA**: (study 5) values uncorrected for multiple comparisons; it only finds a quadratic effect - the linear model is not significant (it reports only values of concatenated bilateral amygdala ROIs - no specific values of R amygdala);<br>**ALE**: no contrast of interest.	MA excluded<br>ALE excluded
15	Ruz et al., 2011	Whole-brain (voxel-wise statistical thresholds $p<.001$, uncorrected; cluster size: 10 or more voxels with cluster correction)	Untrustworthy > trustworthy partners; Trustworthy > untrustworthy partners	**MA:** the results do not display amygdala activity at the whole-brain level;<br>**ALE**: it tests the contrast of interest; whole-brain analysis; values corrected for multiple comparisons (cluster correction);	MA excluded<br>ALE included
16	Said et al., 2009	**MA**: Mask restriction: small volume correction (at $p<.10$)	Negative linear relation with trustworthiness	**MA**: values uncorrected for multiple comparisons (and uncorrected for a small volume of interest at $p<.05$);<br>**ALE**: it tests the contrast of interest; whole-brain analysis; values	MA included<br>ALE included

17	Todorov et al., 2008	**ALE**: Whole-brain (p<.01, uncorrected)<br>Mask restriction: small volume correction (cluster size significant at p<.05: 54mm^3)	Linear relation with trustworthiness	uncorrected for multiple comparisons.<br><br>**MA**: included considering only the conjunction analysis with Engell et al. 2008 data (values corrected for multiple comparisons);<br>**ALE**: not whole-brain, but small volume correction instead.	MA included<br>ALE excluded
18	Tsukiura et al., 2013	Whole brain - voxel level: p<.001 and cluster level: p<.05, using mask restriction (small volume correction) for insula, amygdala, and medial temporal lobe	Linear increases of activity as a function of an untrustworthy impression (given by facial impression of badness) from untrustworthiness to trustworthiness	**MA**: although the contrast met the inclusion criteria, no available statistical values were available for the negative results;<br>**ALE**: for the regions found, it was not used whole-brain analysis, but small volume correction instead.	MA excluded<br>ALE excluded
19	van Rijn et al., 2012	Mask restriction: small volume correction	Untrustworthy faces > baseline	**MA and ALE**: no contrast of interest.	MA excluded<br>ALE excluded
20	Winston et al., 2002	**MA**: Mask restriction: small volume correction (at p<.05)<br>**ALE**: Whole-brain (p<.001, uncorrected; fusiform gyrus corrected at p<.05 using small volume correction)	BOLD signal to untrustworthy faces	**MA:** values corrected for multiple comparisons;<br>**ALE**: it tests the contrast of interest; whole-brain analysis; values uncorrected for multiple comparisons.	MA included<br>ALE included

Note: MA, meta-analysis, ALE, Activation likelihood estimation; FDR, false discovery rate; ROI, region of interest; R, right; L, left.
