# Supplementary material for: The Role of the Amygdala in Facial Trustworthiness Processing: A Systematic Review and Meta-Analyses of fMRI Studies
Source: PLoS One. 2016 Nov 29;11(11):e0167276. doi: 10.1371/journal.pone.0167276 (PMC5127572; doi:10.1371/journal.pone.0167276)
Supplement: S3 Table — Meta-analysis of effect sizes: population characterization, original values (t-scores and Z-scores), contrasts, type of analysis, p-values and corrections taken from the studies feasible for meta-analysis for the contrast "Untrustworthy > Trustworthy" or correlation with facial trustworthiness scores in the (right) amygdala. (PDF) [file pone.0167276.s005.pdf]

Table S3 - Meta-analysis of effect sizes: characterization of studies and data. Population characterization, original values (t-scores and Z-scores), type of analysis, contrasts, p-values and corrections taken from the studies feasible for meta-analysis for the contrast "Untrustworthy > Trustworthy" or correlation with facial trustworthiness scores in the (right) amygdala.

#	Study	Sample size	Gender	Age range (mean±SD)	t	Z	Whole-brain / ROI-based / Mask restriction	Contrasts tested	p-value	Correction for multiple comparisons
1	Baron et al., 2011	24	15M, 9F	19-29 (23)	4,06		Whole-brain	Untrustworthy > trustworthy faces (pre-learning period)	$p < .001$	Uncorrected
2	Bos et al., 2012	16	16F	- (20.8±2.0)	0,27		Mask restriction: small volume correction	Untrustworthy > trustworthy faces (placebo condition)	$p < .05$	Corrected for a small volume of interest
3	Doallo et al., 2012	12	4M, 8F	20-31 (-)		3.59	Whole-brain	No-Go-Low-Trust [untrustworthy] faces > No-Go-High-Trust [trustworthy] faces	$p < .05$	Corrected: FDR corrected for multiple comparisons for the whole brain
4	Engell et al., 2007	14	9M, 5F	- (22.4)	6.83		Mask restriction: small volume correction	Negative linear modulation correlated with consensus ratings (untrust > trust)	$p < .05$	Corrected for a small volume of interest
5	Freeman et al., 2014 (1)	15<br>4M, 12F<br>(1 was later excluded)		18-35 (21.80)	1,19		ROI / Mask restriction: small volume correction	Linear relation with trustworthiness (experiment 2, supraliminal)	$p < .05$	Corrected for a small volume of interest
5	Freeman et al., 2014 (2)				0,25					

6	Gordon et al., 2009	6	4M, 2F	21-29 (23.7)		-2,10	ROI	Linear model of (faces rated for high) Trusting Behavior (not for the quadratic one)	p <.05	Uncorrected
8	Kim et al., 2012	12	12M	- (23.4±2.13) (3 were later excluded)		2,62	ROI / Mask restriction: small volume correction	Negative correlation with facial trustworthiness at the time of face display	p < .005	Uncorrected
13	Platek et al., 2008	11	4M, 7F	- (-)		2,61	Whole-brain	Negative association with consensus ratings of trustworthiness in self2ethnic faces	p < .005	Uncorrected
12	*Pinkham et al., 2008b*	*12*	*12M*	*18-35 (27.08±3.99)*		*2,41(*)*	*ROI / Mask restriction: small volume correction*	*Untrustworthy > trustworthy faces*	*p < .05*	*Corrected for a small volume of interest*
16	Said et al., 2009	32	17M, 15F	- (22,8±6,8)	2,94		Mask restriction: small volume correction	Negative linear relation with trustworthiness	p < .10	Uncorrected
17	Todorov et al., 2008a	14	7M, 7F	18-27 (-)	2,56		Mask restriction: small volume correction	Negative linear relation with trustworthiness (untrust > trust)	p < .05 (conjunction analysis with data of Engell et al., 2007)	Corrected for a small volume of interest
20	Winston et al., 2002	12	8M, 8F	18-30 (23.3)		4,29	Mask restriction: small volume correction	Untrustworthy > trustworthy faces	p < .01	Corrected for a small volume of interest

Note: CI, confidence interval; m.i., missing information; M, males, F, females; R, right, L, left. (*) this study was not included in the meta-analysis of effect sizes given that statistical values were only provided after the meta-analysis has been performed.
