# Supplementary material for: The Role of the Amygdala in Facial Trustworthiness Processing: A Systematic Review and Meta-Analyses of fMRI Studies
Source: PLoS One. 2016 Nov 29;11(11):e0167276. doi: 10.1371/journal.pone.0167276 (PMC5127572; doi:10.1371/journal.pone.0167276)
Supplement: S4 Table — Subgroups analysis: division into subgroups generated according to methodological components taken from the experimental design, data acquisition and analysis parameters. (PDF) [file pone.0167276.s006.pdf]

Table S4 – Subgroups analysis: division into subgroups generated according to methodological components taken from the experimental design, data acquisition and analysis parameters.

Study	n	t	SeqRM - sequence	SeqRM - TR	SeqRM - TE	ParadExp - form	ParadExp - task	Software	Smoothing	Test	Analysis type and correction
Baron et al., 2011	24	4,06	EPI	2s	30ms	Likert	Impl (SP)	AFNI	6mm	Direct contrast	Whole Brains uncorrected
Bos et al., 2012	16	0,27	PRESTO	16ms	23ms	Cat2	Expl (+Impl)*	SPM	8mm	Direct contrast	ROI small volume corrected
Doallo et al., 2012	12	57,25	EPI	3s	30ms	Likert	Expl (+Impl)	SPM	7mm	Direct contrast	Whole Brain Corrected
Engell et al., 2007	14	6,83	EPI	2s	30ms	Likert	Impl (OP)	AFNI	6mm	Correlation	ROI small volume corrected
Freeman et al.(a), 2014	15	1,19	EPI	2s	35ms	Likert	Impl (SP)	BrainV	8mm	Correlation	ROI small volume corrected
Freeman et al.(b), 2014	15	0,25	EPI	2s	35ms	Likert	Impl (SP)	BrainV	8mm	Correlation	ROI small volume corrected
Gordon et al., 2009	6	-8,07	UNKNOWN	2750ms	30ms	Likert	Impl (OP)	FSL	Unspecified	Correlation	ROI uncorrected
Kim et al., 2012	12	21,60	EPI	2s	30ms	Cat2	Impl (OP)	SPM	6mm	Correlation	ROI uncorrected
Platek et al., 2008	11	20,29	UNKNOWN	2.5s	30ms	Likert	Impl (SP)	FSL	6mm	Correlation	Whole Brains uncorrected
Said et al., 2009	32	2,94	EPI	2s	33ms	Likert	Expl	AFNI	4mm	Correlation	ROI small volume corrected
Todorov et al., 2008	14	2,56	EPI	2s	30ms	Likert	Impl (SP)	AFNI	6mm	Correlation	ROI small volume corrected
Winston et al., 2002	12	115,35	EPI	2.5s	16ms	Likert	Expl (+Impl)	SPM	8mm	Direct contrast	ROI small volume corrected

Legend: *BrainV*, BrainVoyager software; *Cat2*, use of 2 (trustworthy, untrustworthy) or 3 (trustworthy, neutral, untrustworthy) categories for facial trustworthiness categorization into conditions; *Likert*, ratings of trustworthiness are performed using an ordinal scale and all values are considered in the analysis; *mm*; millimeters; *ms*, miliseconds; *Expl*, Explicit task; *Impl*, Implicit task; OP, trustworthiness judgements performed by other participants than those of main task; *ROI*, region of interest analysis; *SP*, trustworthiness judgements performed by same participants as of main task; *TR*, repetition time; *TE*, echo time. *it only analyses the explicit task, and it was therefore included in a separated group.
