# Supplementary material for: The Role of the Amygdala in Facial Trustworthiness Processing: A Systematic Review and Meta-Analyses of fMRI Studies
Source: PLoS One. 2016 Nov 29;11(11):e0167276. doi: 10.1371/journal.pone.0167276 (PMC5127572; doi:10.1371/journal.pone.0167276)
Supplement: S5 Table — (A) Articles selection for the negative correlation between faces and trustworthiness (Untrustworthy > Trustworthy); (B) Articles selection for the positive correlation between faces and trustworthiness (Trustworthy > Untrustworthy). (PDF) [file pone.0167276.s007.pdf]

S5 Table. ALE: characterization of studies and data. (A) Articles selection for the negative correlation between faces and trustworthiness (Untrustworthy > Trustworthy); (B) Articles selection for the positive correlation between faces and trustworthiness (Trustworthy > Untrustworthy).

A

#	Study	Sample size	Gender	Age range (mean±SD)	Contrasts tested	p-value	Correction for multiple comparisons	Nr coordinates
3	Doallo et al., 2012	12	4M, 8F	20-31 (-)	No-Go-Low-Trust faces > No-Go-High-Trust faces	p < .05	FDR corrected for multiple comparisons	18
3	(idem)	12	4M, 8F	20-31 (-)	No-Go-Low-Trust faces > No-Go-High-Trust faces	p < .05	FDR corrected for multiple comparisons	6
4	Engell et al., 2007	14	9M, 5F	- (22.4)	Linear modulation correlated with consensus ratings (untrustworthy > trustworthy)	p < .01	Uncorrected	11
13	Platek et al., 2008	11	4M, 7F	- (-)	Negative association with consensus ratings of trustworthiness in self2ethnic faces	p < .005	Uncorrected	5
15	Ruz et al., 2011	14	8M, 10F	20-31 (-)	Negative linear relation with trustworthiness (untrustworthy > trustworthy)	p < .01	Uncorrected	2
16	Said et al., 2009	32	17M, 15F	- (22.8±6.8)	Untrustworthy > Trustworthy	p < .001	Uncorrected	15
20	Winston et al., 2002	12	8M, 8F	18-30 (23.3)	Untrustworthy > Trustworthy	p < .001	Uncorrected	10

B								
**#**	**Study**	**Sample size**	**Gender**	**Age range (mean±SD)**	**Contrasts tested**	**p-value**	**Correction for multiple comparisons**	**Nr coordinates**
6	Gordon et al., 2009	6	4M, 2F	21-29 (23.7)	Trusting Behavior Linear (Trustworthy > untrustworthy faces)	p < .05	FDR corrected for multiple comparisons	5
7	Killgore et al., 2013	39	22M, 17F	18-45 (29.9±8.6)	Increasing vs Decreasing Trustworthiness (Trustworthy > untrustworthy faces)	_	_	0
13	Platek et al., 2008	11	4M, 7F	- (-)	Positive association between trustworthiness ratings and neural activation using self-morph faces (idiosyncratic) and self2sex faces (idiosyncratic)	p < .005	Uncorrected	2
15	Ruz et al., 2011	18	8M, 10F	20-31 (-)	Trustworthy > untrustworthy faces	p < .001	Uncorrected	1
16	Said et al., 2009	32	17M, 15F	- (22.8±6.8)	Positive linear relation with trustworthiness (Trustworthy > untrustworthy faces)	p < .01	Uncorrected	4
20	Winston et al., 2002	12	8M, 6F	18-30 (23.3)	Trustworthy > untrustworthy faces	p < .001	Uncorrected	2

NOTE: Only whole brain results were considered for this analysis.
