# Supplementary material for: The Role of the Amygdala in Facial Trustworthiness Processing: A Systematic Review and Meta-Analyses of fMRI Studies
Source: PLoS One. 2016 Nov 29;11(11):e0167276. doi: 10.1371/journal.pone.0167276 (PMC5127572; doi:10.1371/journal.pone.0167276)
Supplement: S6 Table — Lateralization of amygdala activation within the 20 articles included in the systematic review. (PDF) [file pone.0167276.s008.pdf]

Table S6 – Contrasts performed and lateralization of amygdala activation within the 20 articles included in the systematic review.

#		Articles with experiments included in the MA	Articles with experiments included in the ALE	linear/quadratic	Contrast	AMY R activation	AMY L activation	Studies included in the amygdala lateralization on R/L test (study #)
1	Baron et al., 2011	x		1. linear<br>2. n.a.	1. trustworthy < untrustworthy (pre-learning phase); (Fig. 3a);<br>2. faces presented with behaviors > faces presented without behaviors (during the learning period)<br>(Table 1)	1. yes;<br>2. no	1. no;<br>2. left parahippocampal/ amygdala	1.
2	Bos et al., 2012	x		1. n.a.<br>2. linear<br>3. linear	1. interaction between testosterone administration and trustworthiness judgment<br>2. untrustworthy > trustworthy (testosterone condition) (since it was null for the L amygdala in (1), they did not do the direct t-test U>T in (2));<br>3. untrustworthy > trustworthy (placebo condition) (since it was null for the L amygdala in (1), they did not do the direct t-test U>T in (3));	1. yes;<br>2. yes<br>3. no	1. no;<br>2. (no tested)<br>3. (no tested)	3.
3	Doallo et al., 2012	x	x	Linear	1. No-Go-Low-Trust faces minus No-Go-High-Trust faces	1. yes	1. no	1.
4	Engell et al., 2007	x	x	Linear	1. Linear Modulation Correlated with Consensus Ratings and Idiosyncratic Judgments (corrected using cluster minimum size=162 mm^3 within bilateral amygdala, p.1511) (Table 1)	1. yes	1. yes	1.

5	Freeman et al., 2014 (*)	x		1. linear (with no differences between average and high-trust faces);<br>2. quadratic;<br>3. linear (with no differences between average and high-trust faces);<br>4. quadratic;<br>5. quadratic;<br>6. linear;<br>7. linear	1. Exp.1 (subliminal only): low-trust faces > average-trust faces (bilateral amygdala ROI);<br>2. Exp.1 (subliminal only): (low-trust + high-trust) > average-trust;<br>3. Exp.1 (subliminal only): low-trustworthy > average trustworthy faces (for separate amygdalae voxels within ROI, test (1))<br>4. Exp.2: quadratic effect-supraliminal;<br>5. Exp.2: quadratic effect-subliminal;<br>6. Exp.2 linear effect-supraliminal;<br>7. Exp.2 linear effect-subliminal	1. yes (bilateral amygdala ROI);<br>2. yes;<br>3. yes<br>4. yes;<br>5. yes;<br>6. no;<br>7. no	1. yes (bilateral amygdala ROI);<br>2. no;<br>3. yes<br>4. yes;<br>5. yes;<br>6. no;<br>7. no	6.,7.
6	Gordon et al., 2009	x	x	Linear	1. Linear model of Trusting Behavior (increased results to trustworthy vs. untrustworthy faces)	1. yes	1. yes	-
7	Killgore et al., 2013	n.r.d.	x(**)	Linear	1. Decreasing trustworthiness > Neutral;<br>2. Increasing trustworthiness > Neutral;<br>3. Increasing trustworthiness > Decreasing trustworthiness	1. yes;<br>2. no;<br>3. no	1. no;<br>2. yes;<br>3. no	3.
8	Kim et al., 2012	x		Linear	1. Negative Correlation with Facial Trustworthiness (table 1)	1. yes	1. no	1.
9	Kragel et al., 2015			Linear	1. Increase with untrustworthiness independent of age (vs. baseline)	1. yes	1. no	-
10	Mattavelli et al., 2012			1. quadratic;<br>2. linear	1. quadratic polynomial and<br>2. linear regressions (section 3.3. and Table 2)	1. yes;<br>2. yes (concatenated R+L)	1. yes;<br>2. yes (concatenated R+L)	-
11	Pinkham et al., 2008a			(main effect)	1. Trustworthiness judgments (vs. Baseline) (within each ROI, Table 2)	1. yes	1. yes	-
12	Pinkham et al., 2008b	n.a.s.(***)		Linear	1. Untrustworthy > trustworthy	1. yes	1. no	1.

13	Platek et al., 2008	x	x	Linear	1. Negative association between trustworthiness ratings and activation in amygdala (consensus ratings of trustworthiness in self2ethnic faces in parahippocampal gyrus/ uncus/ amygdala (p. 3, legend Fig. 1; peak voxel of amygdala in Table 1)	1. yes	1. no	1.
14	Rule et al., 2013			1. quadratic; 2. linear	1. quadratic regressor when controlling for the linear regressor; 2. linear regressor when controlling for the quadratic regressor	1. yes; 2. no (concatenated bilateral amygdala ROIs)	1. yes; 2. no (concatenated bilateral amygdala ROIs)	-
15	Ruz et al., 2011	n.r.d.	x	Linear	1. Untrustworthy > Trustworthy partners; 2. Trustworthy > Untrustworthy partners (Table 1)	1. no; 2. no	1. no; 2. no	1.
16	Said et al., 2009	x	x	1. e 2. linear; 3. quadratic	1. Positive linear relation with trustworthiness; 2. Negative linear relation with trustworthiness; (both uncorrected at p<.05); 3. regions showing a quadratic response to trustworthiness after the variance of linear effects (Fig. 2; Table 3)	1. no; 2. yes; 3. yes	1. no; 2. yes; 3. yes	2.
17	Todorov et al., 2008	x		1. linear; 2. quadratic	1. linear; 2. quadratic; (Fig. 2; Table 2)	1. yes; 2. no	1. yes (but the cluster did no pass the significance criterion adjusted for multiple comparisons); 2. yes	1.
18	Tsukiura et al., 2013	n.r.d.		Linear	1. Linear increases with bad impression of faces (Table 2)	1. no	1. no	1.

19	van Rijn et al., 2012			Linear	Untrustworthy faces > baseline	1. (concatenated amygdala) yes	1. (concatenated amygdala) yes	-
20	Winston et al., 2002	x	x	Linear	contrast of untrustworthy to trustworthy faces (Table 2; Fig. 3a)	1. yes. (right, –18, 0, –24; Z = 4.29; $p < 0.05$, corrected for multiple comparisons across a small volume of interest)	1. yes. (left**,** –16, –4, –20; Z = 3.92; $p < 0.05$, corrected for multiple comparisons across a small volume of interest)	1.

NOTE: n.a., not applicable; n.a.s., not available statistical values; n.r.d., no regions displayed; L, left; R, right; ROI, region of interest; (*) results from subliminal presentations of stimuli were not considered; (**) null findings; (***) this study was not included in the meta-analysis of effect sizes as this result was not available at that time. The last column shows the studies, "(study #)", that were included in a non-parametric chi-squared frequency test to evaluate lateralization of amygdala activation.
