# Supplementary material for: The Role of the Amygdala in Facial Trustworthiness Processing: A Systematic Review and Meta-Analyses of fMRI Studies
Source: PLoS One. 2016 Nov 29;11(11):e0167276. doi: 10.1371/journal.pone.0167276 (PMC5127572; doi:10.1371/journal.pone.0167276)
Supplement: S7 Table — Studies displaying results in amygdala, organized according to ventral-to-dorsal activation (considering X coordinates), study design and linearity (linear, non-linear/quadratic) of response. (PDF) [file pone.0167276.s009.pdf]

Table S7 – Studies displaying results in amygdala, organized according to ventral-to-dorsal activation (considering X coordinates), study design and linearity (linear, non-linear/quadratic) of response.

#	study	amy		coordinates (x,y,z) (TAL)			study design	linearity
14	Rule et al.	L		-24	0	-12	Event-related	Quadratic
5	Freeman et al. (exp.2)	L		-22	-5	-17	Event-related	Quadratic
17	Todorov et al.	L		-21	-2	-10	Event-related	Quadratic
16	Said et al.	L	more dorsal to more ventral (according to x coordinate)	-20,27	-5,76	-12,11	Event-related	Linear
7	Killgore et al.	L		-19,3	-1,79	-14,24	Block-design	Linear
4	Engell et al.	L		-16	-5	-19	Block-design	Linear
20	Winston et al.	L		-16	-4	-20	Event-related	Linear
16	Said et al.	L		-13,89	-7,08	-11,31	Event-related	Quadratic
16	Said et al.	R		15,95	-8,28	-7,86	Event-related	Quadratic
16	Said et al.	R		16,49	-5,21	-12,33	Event-related	Linear
20	Winston et al.	R		18	0	-24	Event-related	Linear
8	Kim et al.	R		18,52	-1,08	-3,62	Event-related	Linear
5	Freeman et al. (b, exp.2)	R		19	-6	-16	Event-related	Quadratic
5	Freeman et al. (a, exp.1)	R		21	-12	-10	Block-design	Quadratic
7	Killgore et al.	R	more ventral to more dorsal (according to x coordinate)	21,36	1,2	-7,86	Block-design	Linear
7	Killgore et al.	R		21,38	-0,49	-9,82	Block-design	Quadratic
4	Engell et al.	R		24	-1	-18	Block-design	Linear
3	Doallo et al.	R		24,22	2,73	-13,97	Event-related	Linear
13	Platek et al.	R		25,09	-4,06	-11,9	Event-related	Linear
17	Todorov et al.	R		26	1	-14	Event-related	Linear
14	Rule et al.	R		27	-3	-18	Event-related	Quadratic
