# Supplementary material for: The Role of the Amygdala in Facial Trustworthiness Processing: A Systematic Review and Meta-Analyses of fMRI Studies
Source: PLoS One. 2016 Nov 29;11(11):e0167276. doi: 10.1371/journal.pone.0167276 (PMC5127572; doi:10.1371/journal.pone.0167276)
Supplement: S8 Table — Categorization of facial trustworthiness judgements. (PDF) [file pone.0167276.s010.pdf]

Table S8 – Categorization of Trustworthiness.

#		*Rating of Trustworthiness:*		
		BEFORE	***DURING***	***AFTER***
1	Baron et al., 2011	**categorical**: “All faces had been previously rated on trustworthiness (Engell et al., 2007). From these ratings, six subsets of eight face images (four highly trustworthy and four highly untrustworthy) were created.” (p. 573)		**ordinal:** “After finishing the fMRI part of the experiment, (..) Participants were (...) instructed to rate all 48 faces on their trustworthiness. (...) The response scale ranged from 1 (very untrustworthy) to 9 (very trustworthy).” (p. 576, Fig. 2)
2	Bos et al., 2012		**categorical**: division according to if whether the faces were rated trustworthy or untrustworthy (during the MR task)	
3	Doallo et al.,2012		**ordinal:** “In a subsequent evaluation task, they were required to rate the trustworthiness of the same faces viewed during the previous Go/No-Go task on a 4-points scale from 1 (‘not trustworthy’) to 4 (‘very trustworthy’).” (Fig. 1-B) (p. 651)	

4	Engell et al., 2007	**ordinal:** “16 participants different from the participants in the behavioral studies) ((Each face was presented at the center of the screen with a question above the photograph ‘‘How trustworthy is this person?’’ and a response scale below the photograph. The response scale ranged from 1 (not at all) to 9 (extremely). The face was presented on the screen until the participant’s response.” (p. 1510)		
5	Freeman et al., 2014	**ordinal:** “In a pretest, raters(N=10) judged the trustworthiness of 300 neutral-affect, male and female faces from the Glasgow Unfamiliar Face Database (GUFD; Burton et al., 2010), normalized for size and luminance, in randomized order on a 7-point Likert scale.”	No rating during task. TASK: experiment 1: passive view; experiment 2: passive view; “Experiment 1 used a backward masking paradigm involving 3 levels of masked facial trustworthiness (low, average, high) [in a block design]; In Experiment 2, we extended the backward masking paradigm to a rapid event-related design that allowed us to test amygdala responsivity to a wider and fully continuous range of facial trustworthiness; 1-back task used to maintain subjects’ attention.”	1) with fMRI PARTICIPANTS: **ordinal:** After the scan, subjects were presented with each of the target faces one at a time in randomized order and rated their trustworthiness from 1 (“not at all”) to 7 (“very much”) using the keyboard. These ratings were used on a subject-by-subject, face-by-face basis to model BOLD responses. (p. 10575);<br>2) ADITIONAL BEHAVIORAL EXPERIMENT with OTHER subjects: (p. 10575 e 10576) - (a) **categorical:** “The procedure was identical to the postscan discrimination tasks of Experiments 1 and 2, except that participants were prompted to judge the target’s trustworthiness (untrustworthy or trustworthy?) rather than gender. (b) **ordinal:** Following this initial phase of the experiment, subjects viewed all targets again in a new randomized order, one at a time, with unlimited exposure (no

				masking), and they were asked to judge trustworthiness along a 6-point Likert scale. We used a 6-point rather than a 7-point scale to be able to dichotomize judgments (0–3 untrustworthy; 4–6 trustworthy), thereby permitting signal detection analysis and a controlling of response bias.”
6	Gordon and Platek et al., 2009	**ordinal**: “The Trustworthiness ratings were performed based on questions to the participants who gave their face for the stimuli (photos) in the fMR task (The trustworthiness measure contained the three trusting behaviour questions from Glaeser et al.’s (2000) measures (GSS). The statements questioned the amount of trusting behaviour a participant engages in and is rated on a 5-point Likert scale (5=more than once a week, 1=less than once a year), for example: How often do you lend money to your friends.” (p. 185)		
7	Killgore et al., 2013	**categorical:** 3 levels: decreasing, increasing, neutral. (Fig. 4, p. 342) ("methods described by Oosterhof and Todorov (2008). Briefly, that group used the computer modeling program to generate 300 neutral faces of European origin, which were then subsequently rated by 29 judges on a 9-point scale of trustworthiness.") (p. 337)	No rating during task. TASK: to make a button press each time a stimulus appeared.	

8	Kim et al., 2012	**categorical:** 3 levels: high, medium, low. (Fig. 3, p. 433) Preliminary experiment: “Twenty-six participants evaluated each face on a 9-point scale (1 = not trustworthy at all, 9 = very trustworthy) shown below each face. For each photograph, we combined the rating results of all participants and obtained a normative (or mean) trustworthiness rating by transforming the results into Z scores and averaging across the 26 subjects. On the basis of the normative ratings of the 355 faces, we selected 60 faces (20 faces in each category) with high, medium, or low trustworthiness, and these 60 faces were used as proposers in the ultimatum game.” (p. 429); "We ran a preliminary experiment to reliably select faces with high, medium, and low trustworthiness from among 355 photographs (black and white) of Korean men. (…) Twenty-six participants evaluated each face on a 9-point scale (1 = not trustworthy at all, 9 = very trustworthy) shown below each face."	TASK: Ultimatum game	
9	Kragel et al., 2014		**ordinal:** 4 levels ('--' '-' '+' '++') (p.7)	

10	Mattavelli et al., 2012	Rating performed by other participants:<br>(1) “The 1000-face photographs were rated for trustworthiness (using 1–7 scales) by six independent raters. From these ratings the 15 highest and 15 least trustworthy male faces and the 15 highest and 15 least trustworthy female faces were selected";<br>(2) These continua of 10 images were then presented in random order and rated for trustworthiness (on a 1–7 low–high trustworthy scale) by 10 raters (5 male, 5 female, mean age=20.4 years, S.D.=0.55) who did not otherwise participate in the study.”		**ordinal:** 7 levels. “Participants were asked to rate on a 7-point scale the trustworthiness (1=very untrustworthy, 7=very trustworthy) and the masculinity–femininity (1=high masculine, 7=high feminine) of the images used in the experiment.” (p. 2208)
11	Pinkham et al, 2008a		Rating during MR task. **categorical**: "individuals made a forced choice of trustworthiness, rating each face as either trustworthy or untrustworthy."	
12	Pinkham et al., 2008b		Rating during MR task. **categorical**: “Functional magnetic resonance imaging (fMRI) was utilized while individuals completed the abbreviated Trustworthiness/ Approachability Task. In this task, individuals viewed 42 grayscale frontal images of faces and made dichotomous decisions regarding the trustworthiness (ie, trustworthy or untrustworthy) of the individual in each photo. This procedure was based on Winston et al. Participants responded by pushing a button corresponding to their	

			rating," (p. 690) "The events comprising each condition (trustworthy and untrustworthy) were parsed according to the idiosyncratic judgments of each participant (ie, the subject's individual responses rather than average ratings or categorization based on stimulus qualities)" (p. 691)	
13	Platek et al., 2008			Rating after MR task. **ordinal**: "Four weeks after scanning, participants rated each face they saw during scanning on trustworthiness (idiosyncratic ratings) (see Engell et al., 2007; Winston et al., 2002). Additionally, we asked an independent group of 40 participants (20 female) to rate all the faces on trustworthiness to obtain consensus trustworthiness ratings (see Engell et al., 2007; Winston et al., 2002)." (p. 3)
14	Rule et al., 2013	**ordinal**. Study 5 – MR task - used ratings of trust behavior from previous study 4 (but "no relationship to impressions of trustworthiness from the target faces"). "Stimulus rating. Fifty undergraduates (72% female; Power 99%) at a different university rated the targets' photos in exchange for partial course credit. Participants rated the photos for trustworthiness, intelligence, and extraversion, among other traits, along 7-point scales anchored at 1 (Not at all X) and 7 (Very X)."		

15	Ruz and Tudela, 2011	**categorical**. No rating task was used: "Rather than learning about the trustworthiness of the emotions of their partners by trial and error, participants were explicitly told whether they could trust their partners or not by means of a symbolic cue."; "Participants, who were explicitly informed of the relation between the partners trustworthiness and the emotion they displayed, had to use the information provided by the cue together with the emotion expressed by their partner to accept or reject the offers before they were presented." ; Triangular and squared black shapes were used as trustworthiness cues, which indicated whether the partner for that trial was either trustworthy or untrustworthy (counterbalanced across participants)" (p.1686); "... the assignment of faces to the trustworthiness conditions was arbitrary and fully counterbalanced across participants in our experiment. Faces were assigned to trustworthy and untrustworthy conditions depending on whether their facial displays of emotions could be trusted or not" (p. 1689)	TASK: (modified) Ultimatum Game task. No rating task was used.	No rating task was used.
16	Said et al., 2009	The rating was done before by different participants.	The rating was done during task in the MR. **ordinal**: "during which subjects rated a series of faces on perceived trustworthiness using button presses and a 4-point Likert scale."	

17	Todorov et al, 2008	The rating was done before by a mathematical model: “we computed the predicted trustworthiness value for each of the 66 faces using the regression model obtained in study 1 (Table 1)). (…) At the second stage of the analysis, we regressed the mean trustworthiness judgments on the four facial features. This regression analysis was based on the mean judgments of the unambiguously male faces, as judged by three independent raters, because we used only male faces in the fMRI study.<br>The four facial features accounted for 29.4% of the variance of trustworthiness judgments. The coefficients of the regression model (Table 1) were used to predict the trustworthiness of a new set of faces used in the fMRI study.”		**ordinal**: The rating was done after the MR task: “After the scanning session, subjects were led to a computer and asked to judge the 66 faces used in the fMRI session on trustworthiness.” “Each face was presented at the center of the screen until the subject responded. The response scale ranged from 1 (Very untrustworthy) to 9 (Very trustworthy).” (p. 122)
18	Tsukiura et al., 2013		**categorical**: “During a block of the encoding phase (Figure 1), participants were randomly presented with 60 male faces with neural expressions one by one and were required to rate the personality goodness for each face based on subjective assessment using an 8-button response box (from 1=very bad to 8=very good). The participants were instructed that the personality goodness of the faces should be rated on the basis of trustworthiness, but not be evaluated on the basis of facial attractiveness.” “The	

			personality impression was categorized into three conditions of Bad (levels 1–3), Neutral (levels 4–5) and Good (levels 6–8).” “Badness-related activity in a subjective impression of trustworthiness was identified with a linear regressor (Bad=3, Neutral=2, Good=1)” (p. 517)	
19	van Rijn et al., 2012	pilot study: “These images were selected from a larger set of images on the basis of trustworthiness and emotional valence ratings given by 36 nonclinical subjects in a separate pilot study.”	**categorical:** In the other eight task blocks, which were preceded by the word ‘trustworthiness’, subjects had to judge whether the faces were trustworthy or untrustworthy. (p. 691)	No ratings were requested after the MR task: "we did not ask participants to also rate the faces with regard to trustworthiness after scanning, as these ratings may be different from during scanning due to repeated presentation and related increased familiarity."
20	Winston et al., 2002			**ordinal:** "To account for individual differences in trustworthiness judgment, we acquired ratings of trustworthiness for each stimulus from each subject after scanning and used these ratings as parametric covariates in our subsequent analysis." "trustworthiness scores (from 1, least trustworthy, to 7)" (p.277) *note*: “a second model was constructed by dividing the events for each subject into three groups by rank score for individual stimuli (that is, the least trustworthy third of faces as one event type, the median third as a second, and the most trustworthy third as a third). This model is used in Figs. 1–3 to demonstrate the direction of BOLD signal

	change with respect to trustworthiness score. Note that statistical inferences are drawn solely from the parametric model described above.” (p. 282)
